# Decoupling Abundance from Centrality: Sexual Dimorphism and Geographical Structuring in the *Hyalomma lusitanicum* Microbiome Network

**DOI:** 10.3390/pathogens15080838

**Published:** 2026-08-12

**Authors:** Francisco J. Márquez, Yordanis Pérez-Llano, Manuel de Rojas, Antonio López-Orta, Ruben Tarifa, Antonio Caruz

**Affiliations:** 1 Department of Animal Biology, Vegetal Biology and Ecology, Faculty of Experimental Sciences, Universidad de Jaén, 23071 Jaen, Spain; yperez@ujaen.es (Y.P.-L.); alorta@ujaen.es (A.L.-O.); rtm00039@red.ujaen.es (R.T.); 2 Department of Microbiology and Parasitology, Faculty of Pharmacy, University of Sevilla, 41012 Sevilla, Spain; derojas@us.es; 3 Department of Experimental Biology, Faculty of Experimental Sciences, Universidad de Jaén, 23071 Jaen, Spain; caruz@ujaen.es

**Keywords:** *Hyalomma lusitanicum*, tick microbiome, sexual dimorphism, network topology, keystone taxa, beta diversity, *Francisella*

## Abstract

Background: The tick microbiome is shaped by environmental filters and host biological traits. Microbial co-occurrence networks offer a powerful framework to map inter-microbial interactions, node topological roles, and community stability beyond standard diversity metrics; however, how tick sex modulates these structural networks remains poorly understood. Methods: We sequenced the V3–V4 variable region of the 16S rRNA gene from 86 adult *Hyalomma lusitanicum* ticks (37 males and 49 females) collected across six ecogeographical localities in Andalusia during 2023. A refined dataset of 5570 high-confidence Amplicon Sequence Variants (ASVs) was analyzed. Alpha and beta diversity patterns were evaluated using PERMANOVA models, and sex-stratified co-occurrence networks were constructed using Spearman’s rank correlations and the Zi-Pi topological framework to assess network complexity and keystone roles. Results: The primary obligate endosymbiont *Francisella* dominated the bacteriome (60–90% relative abundance), followed by *Candidatus Midichloria*. PERMANOVA models confirmed that geographical locality (*R*^2^ = 0.111, *p* = 0.003) and host sex (*R*^2^ = 0.095, *p* = 0.001) exerted strong, independent influences on microbial composition. Female ticks harbored a highly streamlined bacterial profile. Conversely, males exhibited significantly higher alpha diversity (*p* < 0.001), reflecting host-sex physiological and behavioral differences that create a broader ecological niche allowing diverse environmental and transient taxa (*Acinetobacter*, *Sphingomonas*, *Stenotrophomonas*, *Brevundimonas*) to colonize. Network analysis revealed a striking sexual dimorphism: female networks were highly centralized and anchored by a single global hub (*Roseomonas*), whereas male networks lacked global hubs and displayed higher topological complexity, achieving stability through a decentralized architecture sustained by 68 inter-module connectors (compared to 24 in females). Despite these macro-topological differences, a core set of seven connector ASVs was conserved across both sexes. Conclusions: These findings demonstrate that host sex shapes the ecological niche of the tick microbiome and drives distinct network topological strategies to maintain community stability, providing new insights into the structural ecology of tick–microbe interactions.

## 1. Introduction

Ticks are obligate blood-feeding arthropods that rank as the primary vectors of animal diseases and the second most important vectors of human pathogens worldwide, following mosquitoes [1,2,3,4]. Due to their unique biology, ticks harbour and transmit a broader diversity of pathogenic agents, including viruses, bacteria, protozoa, and fungi, than most other arthropod groups, generating significant global health concerns [5,6,7]. The epidemiology of tick-borne pathogens is deeply intertwined with the tick life cycle, which enables the acquisition of microorganisms from vertebrate hosts and their maintenance across developmental stages (transstadial transmission) or their exchange between ticks feeding simultaneously on the same host (co-feeding transmission) [8,9,10,11].

However, beyond their traditional role as mere biological conduits, ticks are increasingly recognized as complex ecological landscapes where commensal, mutualistic, and pathogenic microbes continuously interact [12,13,14]. Advances in high-throughput sequencing have sparked a “microbiome revolution” [15], challenging traditional genetic frameworks and promoting integrative evolutionary concepts such as the holobiont and the hologenome [16,17,18,19]. Experimental evidence demonstrates that the bidirectional interactions between the microbiota, the tick, and the tick host are crucial: tick saliva facilitates potential pathogen transmission [20], while bacteria can modulate tick gene expression to enhance tick survival [21]. Furthermore, microbial dysbiosis has been shown to decrease reproductive fitness in ticks such as *Dermacentor andersoni* [22] and drastically alter pathogen colonization, such as reducing *Borrelia burgdorferi* establishment in *Ixodes scapularis* [23] or increasing *Babesia microti* transstadial transmission in *Haemaphysalis longicornis* [24,25].

Among the tick species posing significant health risks in southwestern Europe, *Hyalomma lusitanicum* is uniquely important. It is one of the most abundant exophilic ticks in the central and southern regions of the Iberian Peninsula, as well as in North Africa and other Mediterranean countries [26,27,28]. As a three-host tick species, its adults actively quest for hosts in exophilic environments, primarily parasitizing wild rabbits, hares, and wild ungulates, with domestic animals serving as secondary hosts [29,30]. While *H. lusitanicum* primarily parasitizes wild ungulates and lagomorphs, it plays a key role in the amplification and maintenance of zoonotic pathogens of public health concern, with human biting events remaining exceptional [31,32]. Crucially, as documented in the literature, *H. lusitanicum* is a recognized vector for multiple pathogens, including *Coxiella burnetii*, *Theileria equi*, *Babesia* spp., *Anaplasma* spp., and *Borrelia afzelii* [29], and is strongly implicated in the sylvatic cycle of the Crimean-Congo Haemorrhagic Fever (CCHF) virus [33,34].

Despite the medical and veterinary importance of *H. lusitanicum* and the recognized role of tick-associated microbiota, several ecological knowledge gaps persist. Most microbiome studies have relied on laboratory-reared colonies or focused on compositional descriptions from limited geographical settings. Consequently, the broader structural organization and taxonomic co-occurrence patterns of tick microbial communities in wild populations remain poorly resolved [35,36,37]. Understanding how these microbial assemblages organize themselves into structured networks under natural environmental pressures is essential for advancing our ecological interpretation of tick–microbiome systems.

To address this, our study characterizes the bacterial communities of adult questing *H. lusitanicum* ticks collected under natural conditions in the eastern Andalusia, southern Spain. Using 16S rRNA gene amplicon sequencing at the amplicon sequence variant (ASV) resolution, we assessed the composition, diversity, and taxonomic association networks of these microbial assemblages. By doing so, we aim to describe the ecological structure, variability, and functional architecture of the *H. lusitanicum* microbiome in a natural geographic setting characterized by the active circulation of tick-borne pathogens.

## 2. Materials and Methods

**Figure 1:**
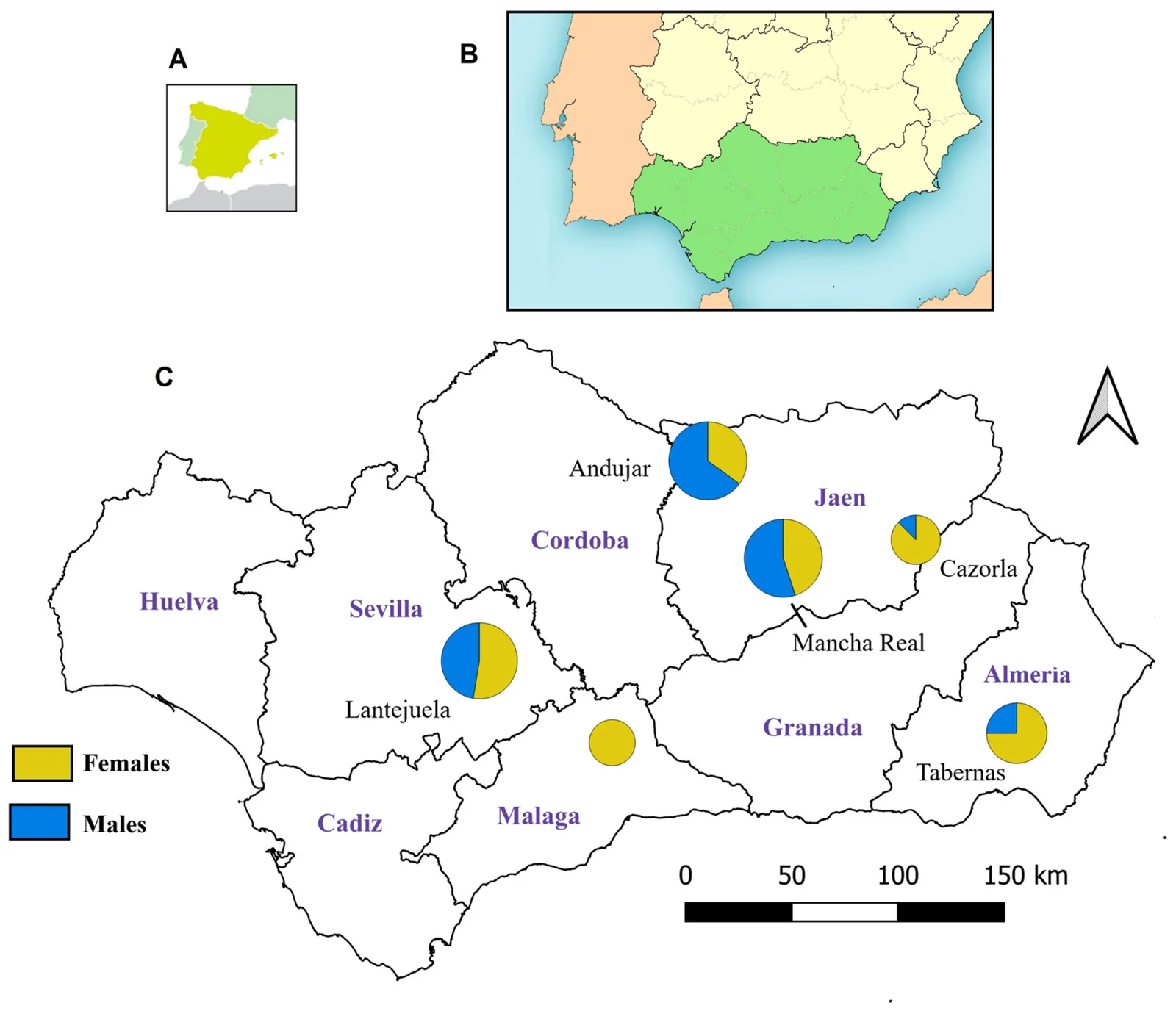
Study area. (**A**) Iberian Peninsula. (**B**) Andalusia. (**C**) *Hyalomma lusitanicum* collection sites.

Questing adult ticks were collected from vegetation across selected localities in Andalusia (southern Spain), encompassing the provinces of Malaga, Sevilla, Almeria, and Jaen, which represent key distribution ranges of *Hyalomma lusitanicum*. Sampling was conducted using the standard flagging technique [38,39] (Figure 1). To ensure a representative characterization of the microbial communities, field collections were strategically timed to coincide with the peak seasonal activity of adult ticks, spanning from spring 2022 to spring 2023.

A total of 86 active adult specimens were selected for subsequent molecular analyses. The selection criteria were strictly based on high vitality and optimal morphological integrity upon arrival, thereby ensuring the quality of the specimens and minimizing potential degradation of the associated microbiota.

The species and sex of the collected ticks were determined through stereomicroscopic examination of external diagnostic features. Taxonomic classification was carried out using the established morphological keys described by Estrada-Peña et al. [40]. Because all analyzed ticks were intact questing adults, species-level identification of *H. lusitanicum* was unequivocally achieved using diagnostic adult structures, including the shape of the genital aperture and scutum punctations in females, and the adanal/subanal plate architecture and conscutal punctation patterns in males.

### 2.1. Isolation and Quality Control of Genomic DNA

To prevent exogenous contamination, all genomic DNA isolation procedures were conducted under rigorous sterile conditions within a dedicated clean workstation. External microbial DNA was minimized through a topical decontamination protocol adapted from Binetruy et al. [41]. Specifically, individual ticks were submerged in a 1% sodium hypochlorite solution for 30 s, followed by three consecutive 1 min rinses in sterile distilled water.

Each specimen was processed independently using pre-sterilized, disposable instruments. To optimize cellular lysis efficiency, ticks were placed in sterile Petri dishes and longitudinally bisected prior to chemical digestion. Total genomic DNA extraction was performed using the DNeasy Blood and Tissue Kit (Macherey-Nagel, Düren, Germany), strictly adhering to the manufacturer’s instructions. To monitor potential background or reagent-derived contamination, mock extraction blanks (containing no biological tissue) were processed in parallel as negative controls. Additionally, laboratory workflows were strictly compartmentalized, maintaining a physical separation between the areas designated for DNA isolation, PCR master mix preparation, and post-amplification cycling.

The concentration of the extracted nucleic acids was determined on a Qubit 3.0 Fluorometer (Thermo Fisher Scientific, Waltham, MA, USA) utilizing the dsDNA High Sensitivity (HS) Assay Kit. Molecular weight distribution and structural integrity were subsequently evaluated via capillary electrophoresis using a Fragment Analyzer (Advanced Analytical Technologies, Inc. [AATI-Agilent], Santa Clara, CA, USA) equipped with the Genomic DNA 50 kb Kit. Downstream 16S rRNA gene library preparation and high-throughput sequencing were restricted exclusively to extracts that satisfied these stringent quality and concentration thresholds.

### 2.2. 16S rRNA Amplicon Library Preparation and High-Throughput Sequencing

The generation of amplicon libraries and subsequent high-throughput sequencing were performed at the IPBLN Genomics Facility (CSIC, Granada, Spain) utilizing a standardized two-step PCR amplification strategy. The hypervariable V3–V4 region of the bacterial 16S rRNA gene was targeted for microbiome profiling. Initial gene-specific amplification was conducted in triplicate for each sample, utilizing 15 ng of template genomic DNA in a final reaction volume of 10 µL. The locus-specific primers V3V4fw (5′-CCTACGGGNBGCASCAG-3′) and V3V4rev (5′-GACTACNVGGGTATCTAATCC-3′) [42] were synthesized to include Nextera overhang molecular adapters and were deployed at a final concentration of 0.2 µM. Reactions were driven by 1× KAPA HiFi HotStart ReadyMix DNA Polymerase (Roche Diagnostics, West Sussex, UK). To maximize diagnostic sensitivity for bacterial target DNA, peptide nucleic acid (PNA) oligonucleotide clamps were incorporated into the master mix to selectively repress the co-amplification of host-derived mitochondrial and plastid 16S rRNA sequences [43].

The thermal cycling profile for the first PCR stage was initiated with a denaturation step at 95 °C for 3 min, followed by 25 cycles of 95 °C for 30 s, PNA clamping at 75 °C for 10 s, primer annealing at 55 °C for 30 s, and elongation at 72 °C for 30 s, concluding with a final extension at 72 °C for 5 min. Following amplification, the triplicate reactions for each sample were pooled. Successful library generation was verified via 1.8% (*w*/*v*) agarose gel electrophoresis. Negative controls, including both extraction blanks and PCR non-template controls, were processed in parallel through all analytical steps and consistently demonstrated the absence of visible bands or amplification artifacts. The pooled amplicons were subsequently purified using the NucleoMag NGS Clean-up and Size Select Kit (Macherey-Nagel).

In the second PCR stage, unique dual combinatorial indices and standard Illumina sequencing adapters were appended to the purified templates using the Nextera XT v2 Index Kit (Illumina, Inc., San Diego, CA, USA). The indexing thermal profile consisted of an initial denaturation at 95 °C for 3 min, followed by 8 cycles of 95 °C for 30 s, 55 °C for 30 s, and 72 °C for 30 s, with a final extension at 72 °C for 5 min. The resulting indexed amplicons were validated again on a 1.8% (*w*/*v*) agarose gel and subjected to a final purification round with the NucleoMag NGS Clean-up and Size Select Kit (Macherey-Nagel). Nucleic acid concentrations were determined using a Qubit fluorometer (Thermo Fisher Scientific). Finally, the purified libraries were pooled in an equimolar ratio, and the structural integrity and fragment size distribution of the final library mix were evaluated on a Bioanalyzer High Sensitivity (HS) DNA chip. Following dilution and denaturation protocols in accordance with Illumina guidelines, high-throughput sequencing was executed on an Illumina MiSeq platform using the MiSeq Reagent Kit v3 (600 cycles) to generate 275 × 2 nt paired-end reads.

### 2.3. Sequence Processing and Microbiome Data Analysis

The bioinformatic processing of raw paired-end FASTQ reads was executed within the R environment [44,45] utilizing the **DADA2** pipeline [46]. Quality control, artifact removal, and low-quality tail trimming were conducted via the filterAndTrim function, establishing a maximum expected error threshold (maxEE) of 2.5 per read. Truncation lengths were intentionally omitted to maximize sequence overlap during the subsequent assembly. After resolving error rates through self-consistent machine learning (selfConsist = TRUE), dereplicated reads were subjected to amplicon sequence variant (ASV) inference via the core Dada algorithm leveraging the pseudo-pooling strategy (pool = “pseudo”). Paired reads were assembled using a conservative minimum overlap of 5 bp, discarding unmerged sequences. Chimeric structures were systematically purged using the de novo bimera checker (removeBimeraDenovo), followed by a secondary verification step using the FindChimeras function from the **DECIPHER** package [47] to yield the final, polished ASV dataset.

An ASV abundance matrix was generated from the clean reads. To mitigate the influence of potential sequencing artifacts or low-confidence background noise, ASVs with an aggregate relative abundance below 0.01% within individual samples were excluded. Taxonomic assignment of representative ASV sequences was performed using the Naïve Bayesian classifier embedded in DADA2 against the SILVA reference database (version 138.2) [48]. When required to resolve unclassified or ambiguous deep-branching assignments, representative sequences were evaluated against the NCBI GenBank database using the BLAST algorithm (v.2.17.0).

Downstream ecological and statistical parsing was handled using the **phyloseq** package [49]. Alpha diversity profiles were estimated based on three complementary metrics: Observed ASV richness, the Shannon diversity index and Pielou’s evenness. Variations in alpha diversity across the different ecological sampling sites (localities) were tested using non-parametric Kruskal–Wallis tests, resolved by Dunn’s post hoc tests with Bonferroni corrections for multiple comparisons. To assess intra-specific dimorphism driven by host sex within *H. lusitanicum*, differences in Shannon diversity indices between male and female cohorts were statically evaluated via non-parametric Wilcoxon rank-sum tests.

To model community composition variation, a distance matrix was constructed using Bray–Curtis dissimilarities based on relative abundances. Beta diversity ordination spaces were subsequently visualized by Principal Coordinate Analysis (PCoA) derived from the calculated Bray–Curtis distances. Prior to multivariate hypothesis testing, the homogeneity of group dispersions across sexes and localities was validated through the *betadisper* function. Main and interaction effects of geographical locality and host sex on the *H. lusitanicum* microbiome structure were then determined through Permutational Multivariate Analysis of Variance (PERMANOVA) using the *adonis2* function within the **vegan** package [50] across 999 permutations. Pairwise breakdowns among geographical sites were subsequently resolved via the *pairwise.adonis2* wrapper [51].

Fine-scale taxonomic distribution patterns were characterized by agglomerating ASV counts to the Genus level using the *tax_glom* function. To decipher shifts in compositional patterns between sexes, the top 50 most abundant bacterial taxa were isolated. Mean relative abundances were computed and statistically compared across male and female hosts, utilizing a log_10_ logarithmic scale transformation via **ggplot2** [52] to ensure the simultaneous and proportional visibility of both dominant symbionts and low-abundance transient genera.

The core microbiome of *H. lusitanicum* was mapped out using the **microbiome** package [53]. Core taxa were filtered through a dual-parameter constraint, requiring a minimum intra-sample relative abundance of ≥0.01% and a persistent cohort prevalence of ≥40%. To establish an empirically optimized baseline for future ecological network modeling and minimize the topological distortion caused by transient or accidental bacteria, a structured network sensitivity framework was designed. *H. lusitanicum* datasets were partitioned by sex and locality metadata. For each sub-cohort, the absolute prevalence of every ASV was monitored along a strictness gradient spanning threshold from 5% to 30%. By identifying structural plateaus and mathematical inflection points in taxonomic decay profiles, monitored and plotted using **ggplot2**, a final optimized prevalence filter of 20% was established to define the final core node matrix reserved for downstream topological co-occurrence network construction.

### 2.4. Microbial Co-Occurrence Network Analysis

To investigate the taxonomic association patterns and structural organization of the bacterial communities associated with *Hyalomma lusitanicum*, a comparative, sex-stratified network-modeling approach was implemented. To ensure methodological robustness and account for potential mathematical artifacts inherent to high-throughput sequencing datasets—such as compositionality biases and closure effects driven by dominant endosymbionts—networks were constructed for both male and female cohorts using two independent mathematical frameworks: non-parametric Spearman’s rank correlations and the composition-aware Sparse Correlations for Compositional Data (SparCC) algorithm [54].

All computational workflows, statistical evaluations, and network visualizations were executed within the R environment (v4.6.0) [44], using the RStudio (v.2025.09.02) integrated development environment [45]. Specific network architectures, topological metrics, and graphical renderings were resolved using the specialized R packages **Hmisc** [55] **NetCoMi** [56], **igraph** [57], **ggraph** [58], and **qgraph** [59].

#### 2.4.1. Sex-Stratified Rank Co-Occurrence Networks (Spearman)

To evaluate the structural architecture and ecological cohesion of the microbiome beyond simple node degree, we implemented a topological cartography analysis based on the Z_i_-P_i_ framework. Rather than assessing metabolic functions, this approach categorizes individual taxa according to their topological roles within and between network modules (e.g., connectors and network hubs). This enables us to identify key structural actors that maintain community stability and facilitate inter-module communication across *H. lusitanicum* sexes.

Microbial co-occurrence networks were constructed separately for male and female *H. lusitanicum* cohorts. To limit the influence of transient or low-frequency taxa and mitigate spurious correlations associated with zero-inflated compositional matrices, the Amplicon Sequence Variant (ASV) abundance dataset was filtered using a strict prevalence threshold (PT = 0.20), retaining only taxa detected in at least 20% of the samples within each respective sex group. Pairwise mathematical associations among the filtered ASVs were calculated via Spearman’s rank correlation using the **Hmisc** package. To ensure high stringency and minimize false-positive edges, correlations were filtered using a strict conservative threshold, retaining only edges with an absolute correlation coefficient of |ρ| ≥ 0.65 and a highly significant nominal *p*-value of *p* < 0.05. The resulting filtered adjacency matrices were modeled as undirected, weighted networks using **igraph**.

Global topological properties—including the total number of nodes and edges, network density, modularity, average clustering coefficient, and network diameter—were computed to compare the structural complexity between male and female microbiomes. At the node level, centrality was evaluated through strength and betweenness metrics.

To resolve the specific ecological roles of individual ASVs beyond global descriptors, each node was mapped into the Guimerà–Amaral functional-cartography space [60] implemented via **ggraph**. This space was defined by calculating each node’s within-module degree (Z_i_; a Z-score measuring intramodular connectivity) and its participation coefficient (P_i_; measuring the evenness of a node’s edges across different modules). Based on the universal thresholds established by Guimerà and Amaral [60], nodes were categorized into four topological roles: peripheral nodes (Z_i_ ≤ 2.5, P_i_ ≤ 0.62), connectors (Z_i_ ≤ 2.5, P_i_ > 0.62), module hubs (Z_i_ > 2.5, P_i_ ≤ 0.62), or network hubs (Z_i_ > 2.5, P_i_ > 0.62) (Table A1). Crucially, to identify whether key topological positions were occupied by dominant symbionts or rare biosphere components, the functional cartography profiles were evaluated by plotting the Z_i_ and P_i_ coordinates directly against the mean relative abundance of each bacterial taxon.

#### 2.4.2. Composition-Aware Network Validation and Cross-Method Comparison (SparCC)

To cross-validate the rank-based co-occurrence patterns and mathematically correct for the high abundance leverage exerted by primary endosymbionts, independent composition-aware networks were constructed at the genus level for both male and female ticks using the SparCC algorithm via the **NetCoMi** package. Empirical pseudo-*p*-values for the SparCC interactions were calculated through a bootstrap approach utilizing 1000 iterations. To maintain absolute methodological alignment with the Spearman framework, SparCC edges were filtered using an identical strict constraint, retaining only statistically significant interactions (*p* < 0.05) with an absolute correlation coefficient of |r| ≥ 0.65.

To ensure the strict reproducibility and structural robustness of the sex-specific co-occurrence networks, a two-step algorithmic anchoring protocol was implemented. First, prior to computingthe Spearman’s rank correlation coefficients, the filtered OTU matrices were deterministically sorted in alphabetical order based on their ASV identifiers. This column-sorting step eliminated any potential variability in the initial node sequence transferred to the graph objects. Second, because community detection via the Louvain algorithm (*cluster_louvain* function in **igraph**) relies on a heuristic greedy approach that is inherently sensitive to node ordering and random tie-breaking, a local pseudo-random number generator seed was explicitly fixed (set.seed(123)) immediately preceding the modularity optimization for both male and female datasets. These dual constraints framework guaranteed that the resulting module boundaries, within-module connectivity (Z_i_), and participation coefficients (P_i_) remained mathematically identical across independent computational runs, ensuring full transparency and reproducibility of the topological roles.

Global and node-level topological metrics (nodes, edges, diameter, modularity, average degree, strength, clustering coefficient, and density) were computed using **igraph** for the SparCC networks. Finally, a comprehensive cross-method comparison was performed, contrasting the network topologies obtained for males and females under both the Spearman and SparCC frameworks. This dual-algorithmic approach allowed us to evaluate the mathematical stability and biological robustness of the *H. lusitanicum* microbial interactions across different statistical assumptions. The final composition-aware architectures were graphically rendered and compared using **qgraph**.

## 3. Results

The analyzed sample included 86 ticks belonging to *H. lusitanicum* (37 males and 49 females), which were captured in six localities of Andalusia during 2023 (Figure 1). Their distribution across the six sampled localities is detailed in Table 1.

High-throughput sequencing generated a total of 3,850,473 high-quality non-chimeric reads, with an average of 44,772.9 sequences per sample (SD = 24,933.1). The generated 16S rRNA amplicons exhibited a sequence length ranging from 350 to 437 bases (b), with an average length of 415.5 ± 10.89 b, confirming the successful and specific amplification of the targeted V3–V4 variable region. An ASV abundance table was constructed from these processed reads, containing initially 8799 ASVs. To eliminate potential spurious or low-confidence taxa, ASVs representing less than 0.01% of total reads within a sample were strictly removed, resulting in a final dataset of 5570 high-confidence ASVs and a total of 3,794,858 remaining reads.

### 3.1. Alpha and Beta Diversity

**Figure 2:**
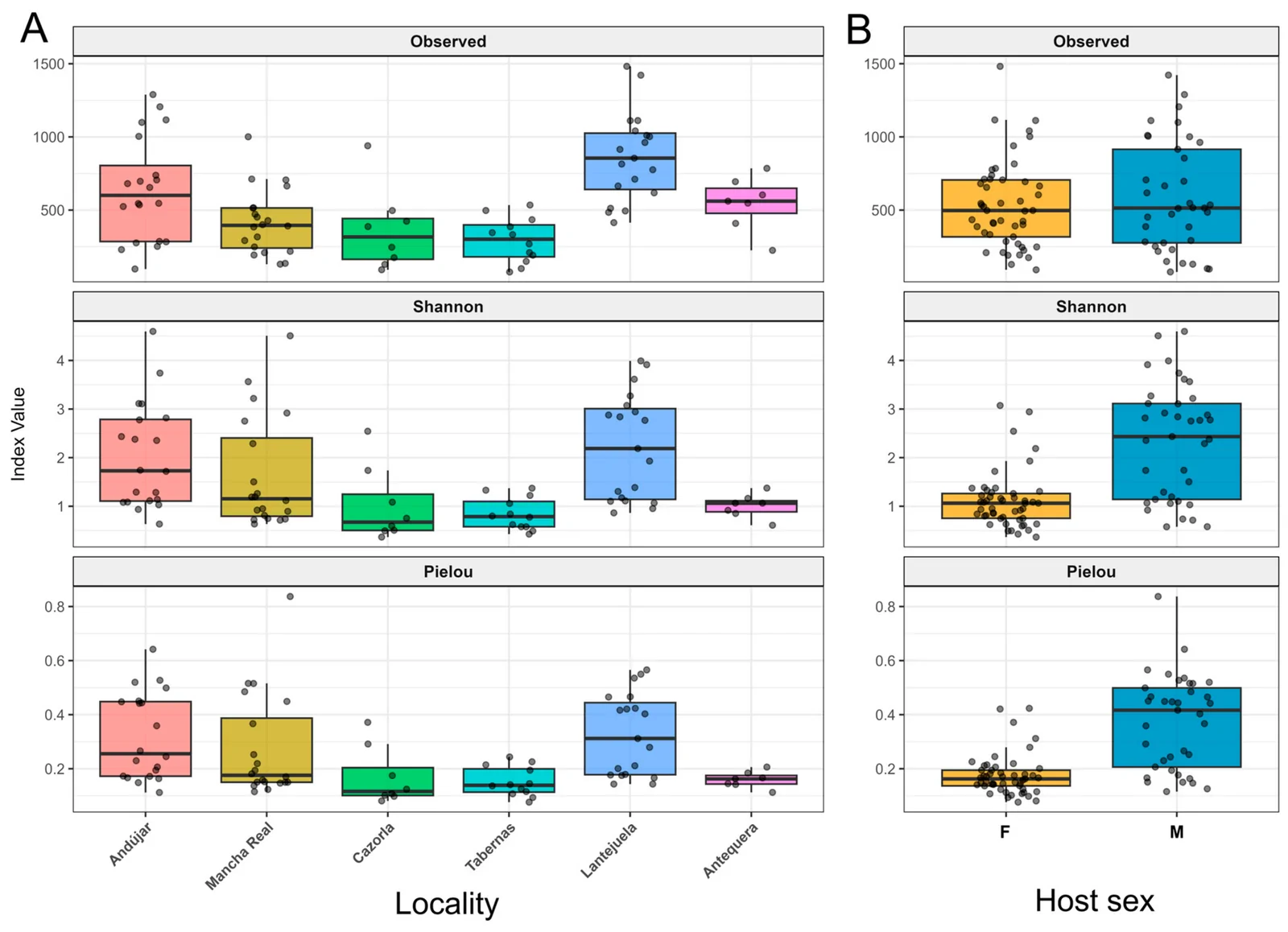
Alpha diversity profiling of the *Hyalomma lusitanicum* microbiota across ecological localities and host sexes. Diversity metrics were calculated based on Observed ASVs, Shannon index, and Pielou’s evenness. (**A**) Comparative boxplots illustrating alpha diversity variations across the six sampled localities. (**B**) Comparative boxplots displaying diversity indices stratified by host sex (Females, n = 49; Males, n = 37). Boxes represent the interquartile range (IQR), with the internal horizontal line indicating the median; individual data points (jittered dots) represent the alpha diversity values of each tick.

Both ecological geography and host traits were identified as key drivers of the bacterial community structure in questing *H. lusitanicum*. All three alpha diversity metrics (Observed ASVs (richness), Shannon Index, and Pielou’s Evenness) revealed consistent and significant trends (Figure 2). While geographical locality exerted a notable influence, with Andujar and Lantejuela generally exhibiting the highest diversity across all indices compared to other sites, the most profound driver of community structure was tick sex. Strikingly, male ticks possessed significantly higher species richness, more balanced relative abundances (higher evenness), and consequently greater overall Shannon diversity than females, highlighting a robust sexual dimorphism in tick microbiome composition. Alpha diversity, measured by the Shannon index, exhibited highly significant differences between males and females (Wilcoxon rank-sum test, *p* < 0.001).

**Figure 3:**
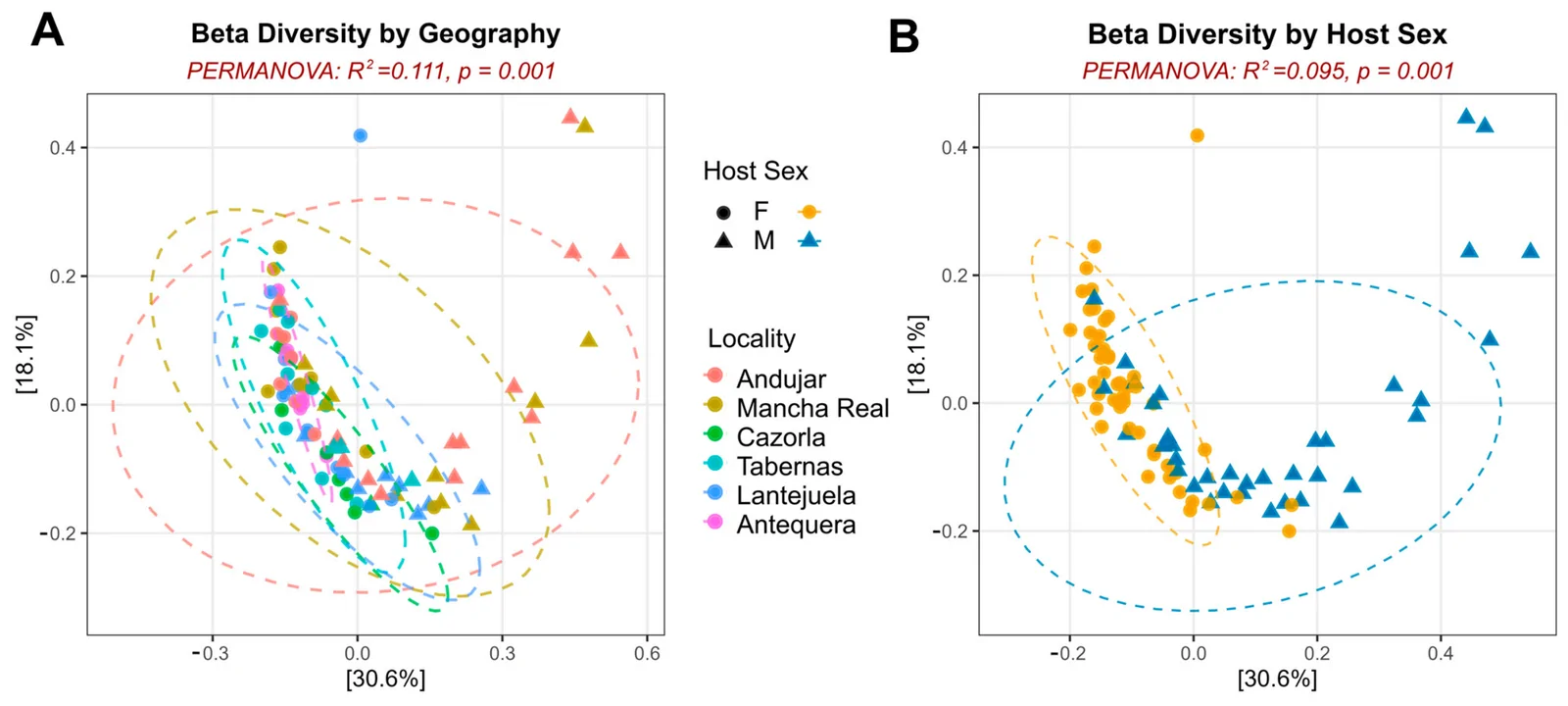
Beta diversity of the *H. lusitanicum* microbiota. Principal Coordinates Analysis (PCoA) plots based on Bray–Curtis’s dissimilarity. (**A**) Ordination plot representing samples grouped by geographical sampling locality. (**B**) Ordination plot representing samples stratified by host sex (Females: orange circles; Males: blue triangles). Dashed ellipses denote the 95% confidence interval for each categorical group. Principal coordinate axes indicate the percentage of variance captured by the ordination.

Regarding beta diversity, Principal Coordinates Analysis (PCoA) based on Bray–Curtis’s dissimilarities revealed distinct microbial compositional profiles associated with sampling location and host sex (Figure 3). Prior to running PERMANOVA models, homogeneity of multivariate dispersion was evaluated using *betadisper* (Table A2). Sampling locality satisfied the assumption of homogeneous dispersion (*F*_21,64_ = 1.32, *p* = 0.248), confirming that location-specific differences reflect true spatial shifts in community centroids. In contrast, host sex exhibited significant heteroscedasticity (*F*_1,84_ = 31.55, *p* < 0.001), driven by higher compositional variability in males relative to females. To evaluate the independent and interactive effects of host sex and environmental geography while accounting for dispersion, a multifactorial PERMANOVA model was constructed. The global model accounted for 24.3% of the total bacterial community variation (*R*^2^ = 0.243, *p* = 0.001). When partitioning this variance, the ecological sampling site exerted a strong driver effect, explaining 11.1% of the total variation (*R*^2^ = 0.111, *p* = 0.001) (Figure 3A). Host sex also significantly shaped the microbial profiles, accounting for 9.5% of the variance (*R*^2^ = 0.095, *p* = 0.001) (Figure 3B), reflecting a combination of structural shifts and gender-specific dispersion patterns. Notably, the interaction between host sex and locality was not statistically significant (*R*^2^ = 0.037, *p* = 0.470). This indicates that the microbial divergence observed between sexes remains consistent across geographical capture sites.

Pairwise Wilcoxon rank sum exact tests with Benjamini–Hochberg adjustment revealed specific geographical divergences in alpha diversity. Notably, the bacterial richness in Cazorla differed significantly from Andujar (*p* = 0.0019) and Antequera (*p* < 0.001). Conversely, localities such as Andujar and Antequera exhibited homogeneous microbiome diversities (*p* = 0.53), suggesting similar ecogeographical constraints shaping the tick microbiome in these specific sites.

### 3.2. Selected Genus Abundance

**Figure 4:**
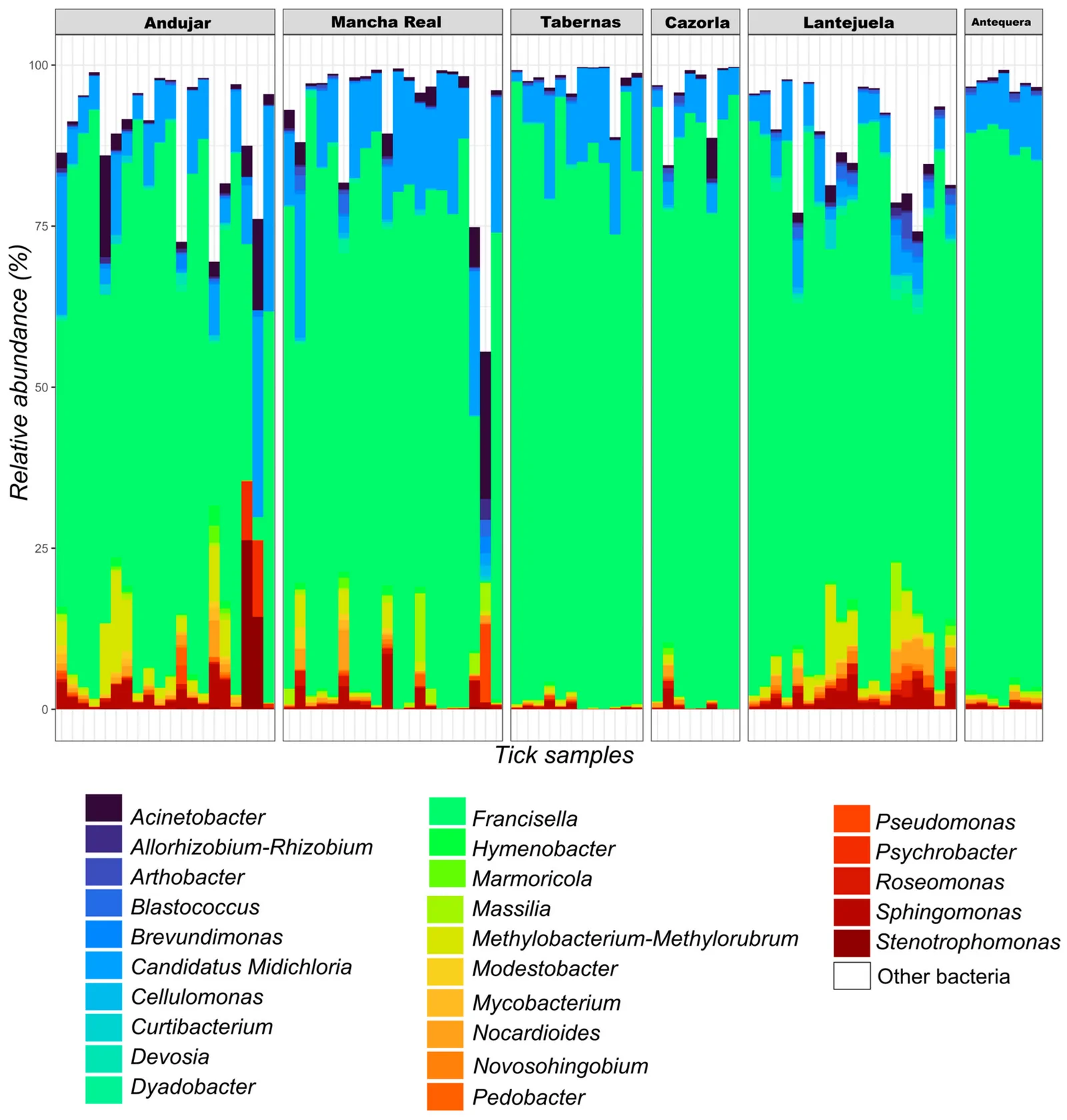
Relative abundance of bacterial genus across individual tick samples by localities. Each vertical bar represents the bacteriome composition of a single tick individual. Taxa are color-coded at the family level. To enhance clarity, only the most 25 frequent genera are shown individually; all remaining taxa are grouped into the “Others” category. Taxonomic assignments were performed against the SILVA database (v.138).

**Figure 5:**
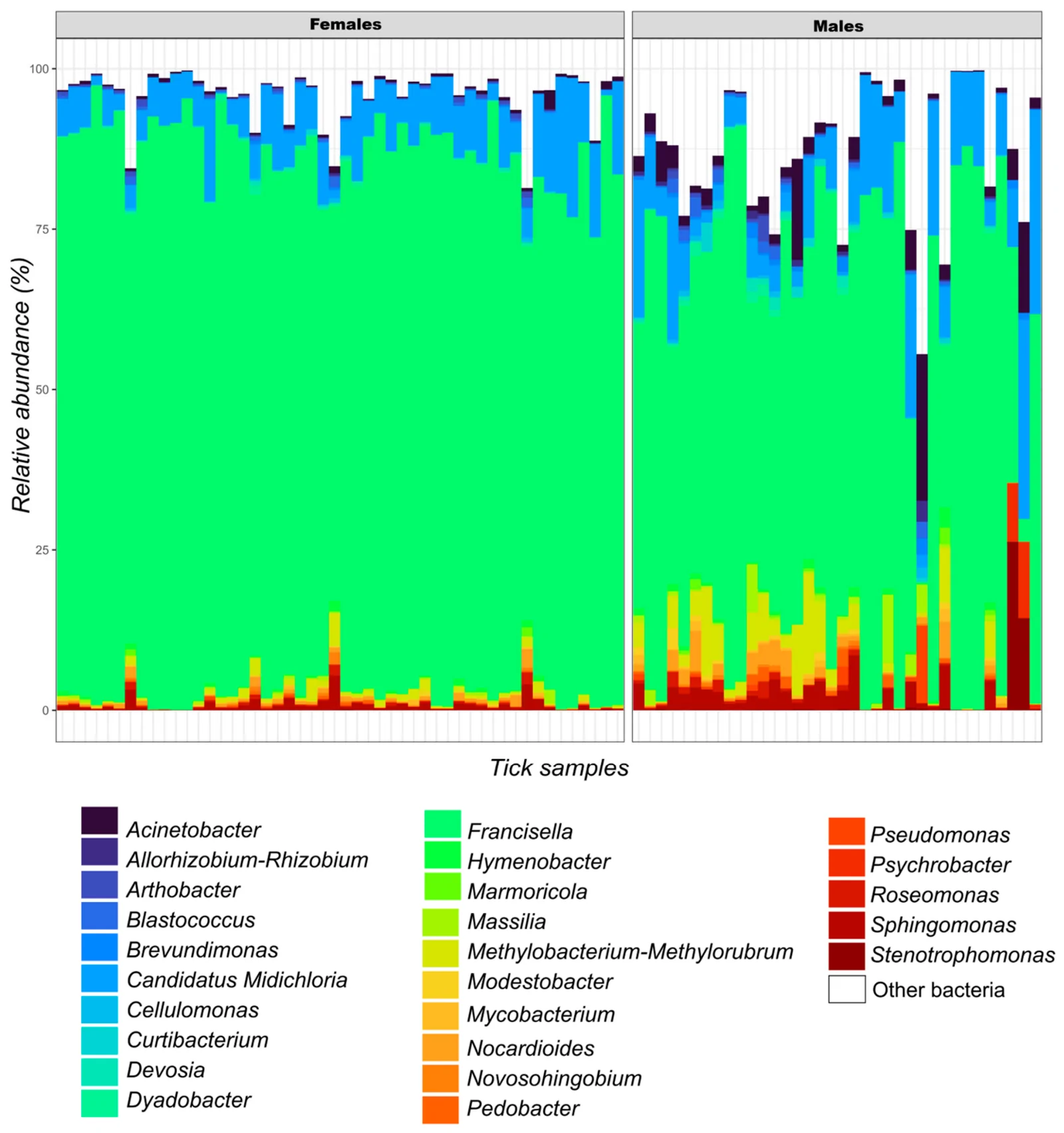
Relative abundance of bacterial genera across individual *H. lusitanicum* tick samples, stratified by host sex. Each vertical bar represents the bacteriome composition of a single individual. Taxonomic identification and color-coding are provided at the genus level (Top 25 most frequent genera); low-abundance taxa are implicitly captured in the upper baseline blank space. Assignments were performed against the SILVA database (v.138).

The observed patterns of abundance were consistent across both geographical localities (Figure 4) and host sexes (Figure 5), confirming that while geographical origin influences baseline community structure, host sex is a primary determinant of individual microbiome variability.

The taxonomic characterization confirmed the ubiquitous and overwhelming dominance of the genus *Francisella* (light green bars) across all individual ticks and geographical locations (Figure 4). In the vast majority of specimens, *Francisella* constituted more than 60% to 90% of the total bacteriome, validating its role as the primary obligate endosymbiont of *H. lusitanicum* regardless of environmental or local factors [61]. The specific GenBank accession numbers and taxonomic assignments for the *Francisella* sequences resolved in this study are detailed in Table A3.

In tandem, *Candidatus Midichloria* (light blue bars) was identified as the prominent secondary co-symbiont. However, unlike *Francisella*, its distribution exhibited substantial inter-individual and inter-locality fluctuations. While highly prevalent and abundant in several individuals from Andujar, Mancha Real, and Lantejuela, its presence was remarkably reduced or nearly absent in the population from Tabernas.

Interestingly, geographical location exerted a visible effect on the baseline diversity of the companion microbiota. The tick population from Tabernas displayed a highly streamlined and homogeneous microbiome, almost exclusively restricted to the *Francisella*-*Midichloria* consortium. In contrast, localities such as Andujar, Mancha Real, and Lantejuela presented a more diverse influx of transient or environmental bacteria.

Despite the general population trends, sporadic inter-individual anomalies were captured. Notably, a single specimen from Mancha Real exhibited a critical disruption of the standard profile, where *Francisella* abundance dropped below 50%, co-occurring with a massive dysbiotic expansion of *Acinetobacter* (dark purple) and *Brevundimonas* (dark blue). Similarly, isolated individuals in Andujar and Lantejuela showed prominent enrichments of environmental Proteobacteria, such as *Sphingomonas* and *Stenotrophomonas* (red and brown basal bars), highlighting that individual horizontal acquisition of microbiota occurs dynamically in the wild.

### 3.3. Differences Between Male and Female Ticks

Stratification of the taxonomic data by host sex revealed a clear contrast in microbiome stability and individual uniformity. Female ticks exhibited a highly conserved and streamlined bacteriome profile, almost entirely dominated by the *Francisella* and *Candidatus Midichloria* core consortium, with minimal background variation from other bacterial groups (Figure 5, left panel). Conversely, male ticks presented a higher degree of inter-individual heterogeneity (Figure 5, right panel). This male-associated plastic pattern was characterized by a more frequent and substantial contribution of companion bacterial lineages, such as *Acinetobacter*, *Sphingomonas*, *Stenotrophomonas*, and *Brevundimonas*, which accumulated in the basal fraction of the microbiome. While the primary endosymbiont *Francisella* remained foundational in both sexes, these results indicate that the male physiological or behavioral landscape allows for a more permissive colonization or persistence of diverse environmental and transient bacterial taxa compared to the strict, highly uniform microbial profile observed in female hosts.

**Figure 6:**
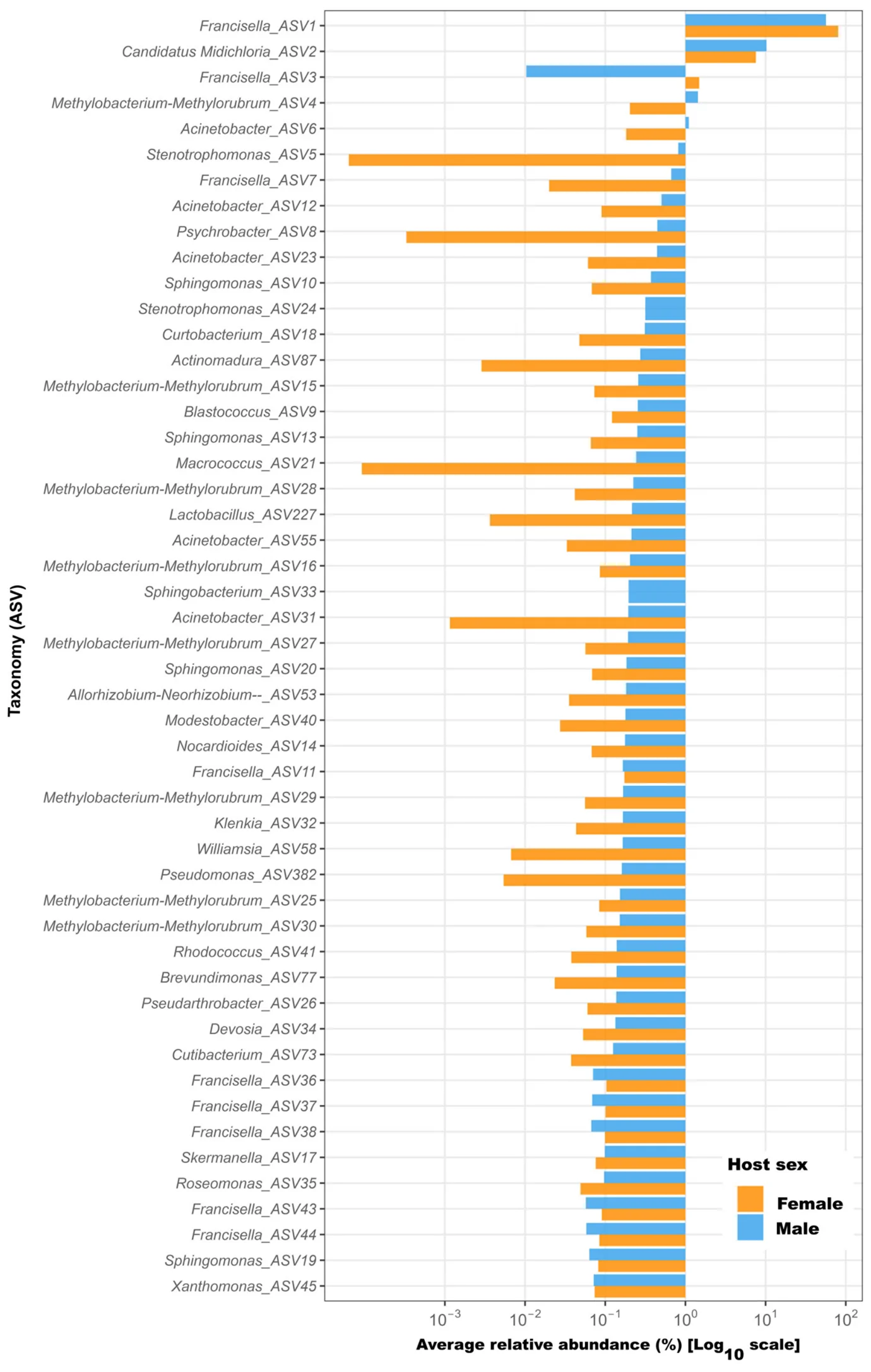
Abundance profiling of the top 50 bacterial Amplicon Sequence Variants (ASVs) contrasted between female and male *H. lusitanicum* ticks. The horizontal axis displays the average relative abundance (%) on a log_10_ scale ranging from 10^−3^ to 10^2^, with the 10^0^ (1%) value serving as the central baseline. Bars extending to the right depict dominant taxa exceeding 1% mean relative abundance (e.g., *Francisella*_ASV1, *Candidatus Midichloria*_ASV2), whereas bars extending to the left represent lower-abundance companion microbiota (<1%). The vertical axis lists the taxonomic identification and specific ASV designation. Colors differentiate host sex, with orange bars representing female ticks (n = 49) and blue bars representing male ticks (n = 37). This comparative layout highlights sexual dimorphism and baseline similarities in the colonization patterns of primary endosymbionts and associated bacterial genera.

The abundance profiling of the top 50 ASVs revealed clear quantitative asymmetries between host sexes (Figure 6). Notably, while *Francisella*_ASV1 (Genbank ascession MW131669 and others detected in *H. lusitanicum*; [62] and *Candidatus* Midichloria_ASV2 (Genbank accession HF568836; [63] established the dominant core microbiota in both sexes, *Francisella*_ASV3 (99.77% similarity with MW1316689) exhibited a strict sex-biased distribution, being prominently detected within the top 50 framework of male ticks while remaining below the inclusion threshold in females. The remaining companion microbiota showed relatively homogeneous distribution patterns between sexes, consisting primarily of low-abundance transient genera fluctuating between 10^−1^ and 10^−3^.

### 3.4. Topological Roles of the Co-Occurrence Networks

To facilitate a seamless interpretation of the dataset and clarify the methodological transition between the global network characterization and the final graphical outputs, a distinction in the correlation thresholds must be highlighted. While the baseline co-occurrence properties and topological metrics were standardly calculated using a uniform threshold of |ρ| ≥ 0.65, the visual representations of the Spearman core networks were intentionally subjected to asymmetric, high-stringency filtering (|ρ| ≥ 0.70 for females and |ρ| ≥ 0.85 for males). This targeted adjustment was implemented exclusively for visualization purposes to mitigate the ‘hairball effect’, which otherwise completely obscured the hyper-dense male microbial co-occurrence network, thereby exposing the high-confidence structural backbone and allowing a mathematically consistent, legible comparison between the core microbiomes of both sexes.

**Figure 7:**
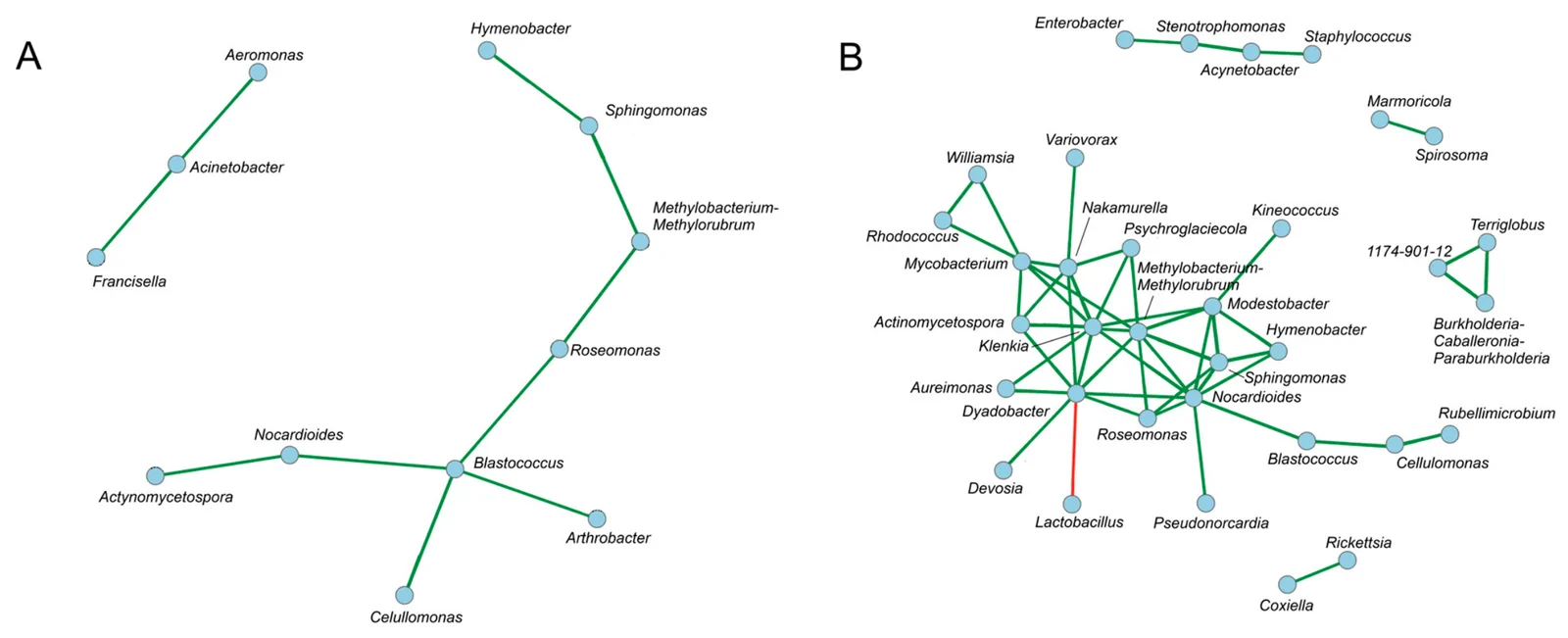
Comparative architecture of SparCC co-occurrence networks in the *H. lusitanicum* microbiome. Integrated analysis of the bacterial community reveals a marked topological divergence based on host sex. (**A**) Female microbiome network, characterized by a centralized structure and discrete interaction channels. (**B**) Male microbiome network, showing a massive, dense, and redundant interconnection core. Nodes represent amplicon sequence variants (ASVs) identified at the genus level. Edges indicate robust statistical interactions. Methodological confidence parameters: internal prevalence threshold PT = 0.2, strict correlation strength |ρ| ≥ 0.65, and false discovery rate corrected *p*-value FDR < 0.05. Sub-networks showing significant inter-bacterial interactions: Green edges represent statistically significant positive correlations (*r* > 0, co-occurrence), whereas red edges represent statistically significant negative correlations (*r* < 0), indicating potential competitive or co-exclusion relationships between bacterial taxa. Notably, in male ticks, a strong negative interaction (red edge) is identified between *Lactobacillus* and *Dyadobacter*, while the remaining depicted taxa exhibit positive interactions (blue edges).

Microbial co-occurrence analysis using the SparCC algorithm revealed a profound sexual dimorphism in the ecological architecture of the *H. lusitanicum* microbiome. The female network (Figure 7A) exhibits a markedly centralized, low-density topology characterized by a “top-down” control structure. In this scenario, most taxa occupy the periphery with low local connectivity, remaining subordinated to the dynamics of a few dominant central components. Conversely, the male microbiome (Figure 7B) presents a diametrically opposed configuration: a highly dense, decentralized, and redundant network. This massive framework of tightly coupled interconnections suggests the presence of complex metabolic consortia and a distributed control system (connector pipeline). This cooperative architecture in males not only optimizes the division of ecological labor within the host but also confers greater resilience and stability to the microbial ecosystem against external perturbations, contrasting with the fragmented and insular system observed in females.

Complementary to the SparCC analysis, the Spearman rank correlation networks, filtered under asymmetric stringency thresholds (|ρ| ≥ 0.65), further substantiate the profound sexual dimorphism in the *H. lusitanicum* core microbiome.

To facilitate the biological interpretation of these complex topologies, node roles were categorized using the Z_i_–P_i_ topological framework, translating abstract mathematical metrics into specific ecological functions within the tick holobiont (summarized in Table A1).

**Figure 8:**
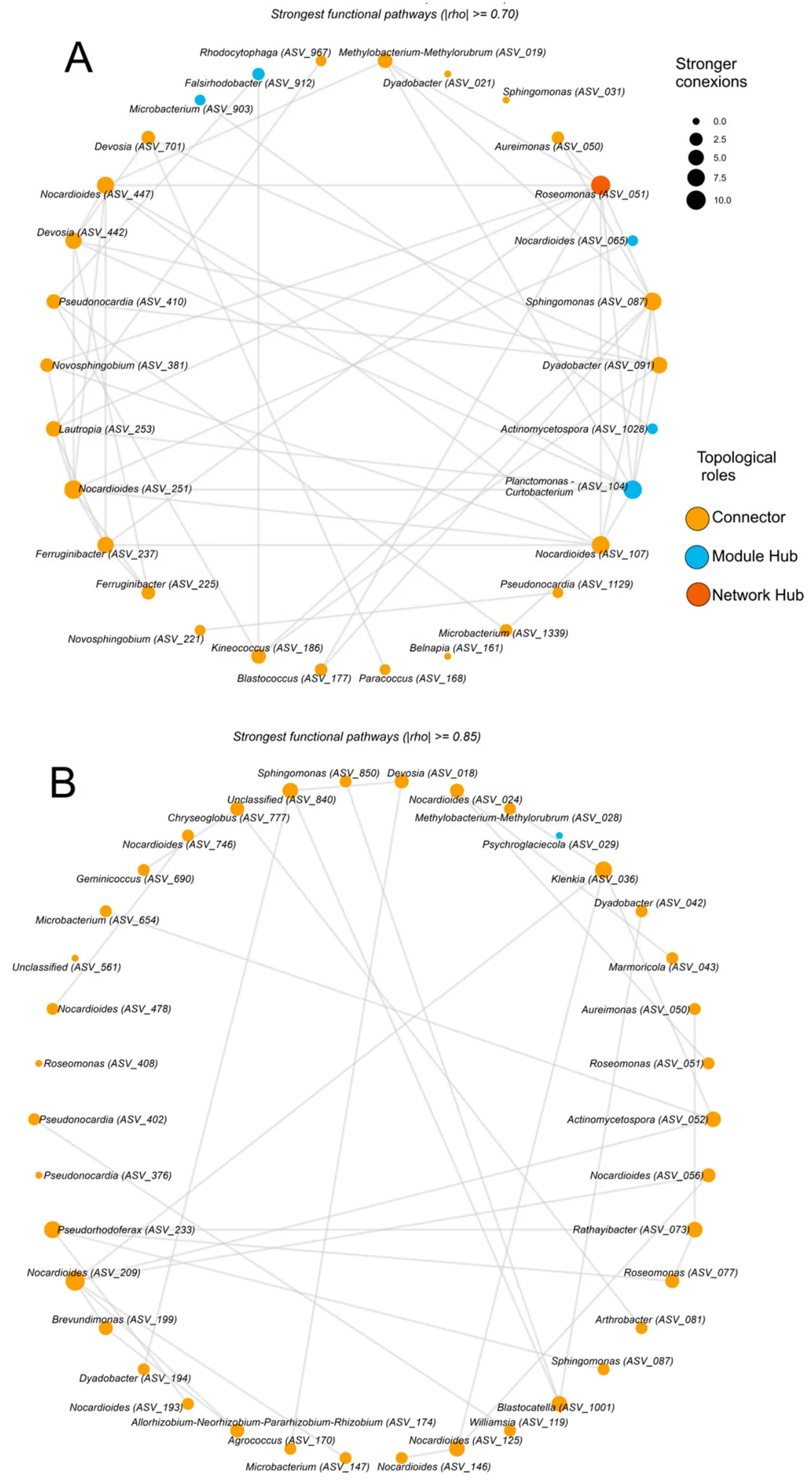
Spearman rank co-occurrence networks illustrating the topological backbone of the *H. lusitanicum* core microbiome. Asymmetric correlation thresholds were applied to resolve and compare the core interactomes while eliminating peripheral density. (**A**) Female core network constructed at a strict correlation threshold of |ρ| ≥ 0.70, highlighting a centralized architecture governed by specific keystone anchors, including the *Roseomonas* (ASV_051) Network Hub and five bacterial ASVs that actuate as Module Hub. (**B**) Male core network constructed under an ultra-strict filtering criterion (|ρ| ≥ 0.85), demonstrating a persistently dense and robustly interconnected backbone comprising functional Connectors and specialized Module Hubs (e.g., *Psychrobacillus* ASV_029). Notably, transitioning from the baseline threshold of |ρ| ≥ 0.65 to the extreme value of |ρ| ≥ 0.85 results in the visual exclusion of the *Sphingomonas* (ASV_008) Module Hub due to the strict mathematical constraints of the filter. Nodes represent amplicon sequence variants (ASVs) assigned at the genus level; node size is proportional to the connectivity degree within the filtered backbone, and color denotes the specific topological role (Network Hub, Module Hub, or Connector). Edges represent strong, statistically significant Spearman correlations. Methodological confidence parameters: internal prevalence threshold PT = 0.2 and FDR-corrected *p*-value < 0.05.

In the female network, out of 384 total nodes and 1578 edges, 30 taxa (7.81%) were classified as structurally influential, with the vast majority of the community remaining as peripherals (Figure 8A). Notably, a single Network Hub was detected exclusively in the female microbiome: *Roseomonas* (ASV_051), positioning this specific taxon as a global moderator mediating community-wide cohesion, complemented by a discrete Module Hubs that harbored 5 bacteria that acted as local coordinators within their respective modules, identified as *Actinomycetospora* (ASV_1028), *Falsirhodobacter* (ASV_912), *Microbacterium* (ASV_903), *Nocardioides* (ASV_065), and *Planctomonas-Curtobacterium* (ASV_104), reflecting a centralized control mechanism where specific core operational taxonomic units drive community cohesion. Furthermore, 24 taxa were classified as Connectors, serving as essential biological bridges across different modules. These connectors comprised a diverse assemblage including representatives from *Aureimonas*, *Belnapia*, *Blastococcus*, *Devosia*, *Dyadobacter*, *Ferruginibacter*, *Kineococcus*, *Lautropia*, *Methylobacterium-Methylorubrum*, *Microbacterium*, *Nocardioides*, *Novosphingobium*, *Paracoccus*, *Pseudonocardia*, *Rhodocytophaga*, and *Sphingomonas*. The persistence of this cross-talk under extreme mathematical stringency underscores a highly coordinated, redundant, and resilient cooperative metabolic engine in males. Conversely, the female network, even at a more permissive threshold (|ρ| ≥ 0.70), displays a more centralized and topographically stratified architecture (Figure 8A).

Conversely, the male network exhibited a substantially denser and more interconnected topology consisting of 492 nodes and 6501 edges, preserving 70 key network-stabilizing actors (14.23%) (Figure 8B). Unlike females, the male network entirely lacked global Network Hubs but possessed two local Module Hubs: *Psychroglaciecola* (ASV_029) and *Sphingomonas* (ASV_008). However, the number of Connectors in the male network expanded drastically to 68 taxa, representing a highly redundant and versatile multicommunity pipeline. Interestingly, a highly conserved topological core was unveiled at the sequence level between both sexes. Seven distinct ASVs consistently maintained their roles as connectors in both networks, including *Aureimonas* (ASV_050), *Belnapia* (ASV_161), *Devosia* (ASV_442), *Dyadobacter* (ASV_021), *Ferruginibacter* (ASV_225), *Novosphingobium* (ASV_381), and *Sphingomonas* (ASV_087).

Even when subjected to an ultra-strict threshold of 0.85 designed to prune peripheral noise, the male core network exhibits a remarkably robust, densely interconnected topological backbone dominated by an extensive array of functional.

Remarkably, two additional key ASVs retained high topological importance across both sexes but dynamically shifted their functional positions: *Roseomonas* (ASV_051) transitioned from a global Network Hub in females to a Connector in males, whereas *Nocardioides* (ASV_065) acted as a local Module Hub in females while functioning as an inter-module Connector in the male network.

**Figure 9:**
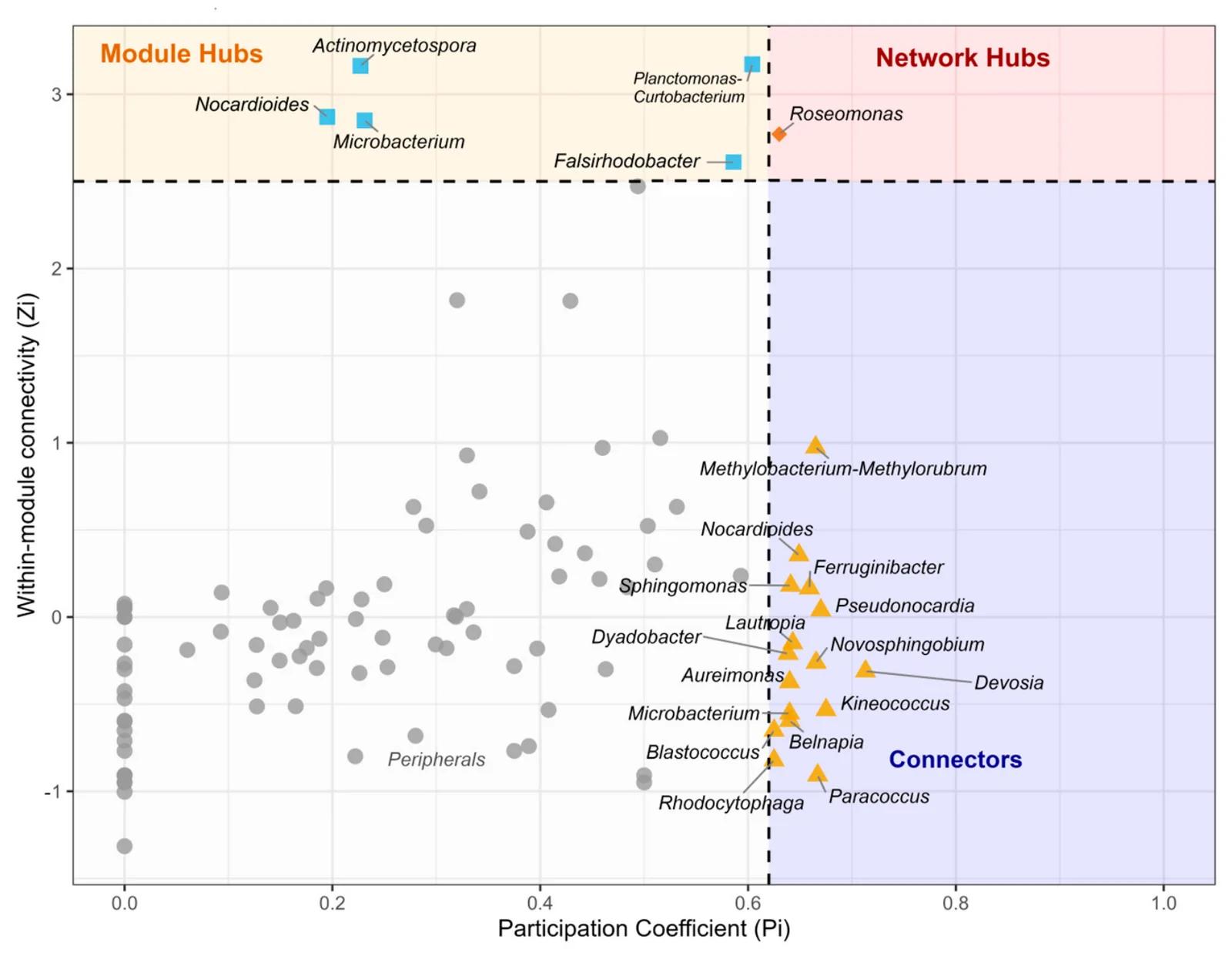
Network topology and topological roles of the bacterial community in female *H. lusitanicum* ticks. The plot displays the distribution of bacterial taxa based on their within-module connectivity (*Zi*) and participation coefficient (*Pi*) values, derived from a Spearman co-occurrence network analysis (Figure 9 and Figure 10). The classification of nodes into topological roles is demarcated by dashed lines at *Zi* = 2.5 and *Pi* = 0.62. Taxonomic labels indicate key bacterial genera acting as connectors (triangles), which facilitate inter-module communication within the male tick microbiome. Peripheral nodes (circles) represent taxa with localized connectivity patterns within their respective modules.

**Figure 10:**
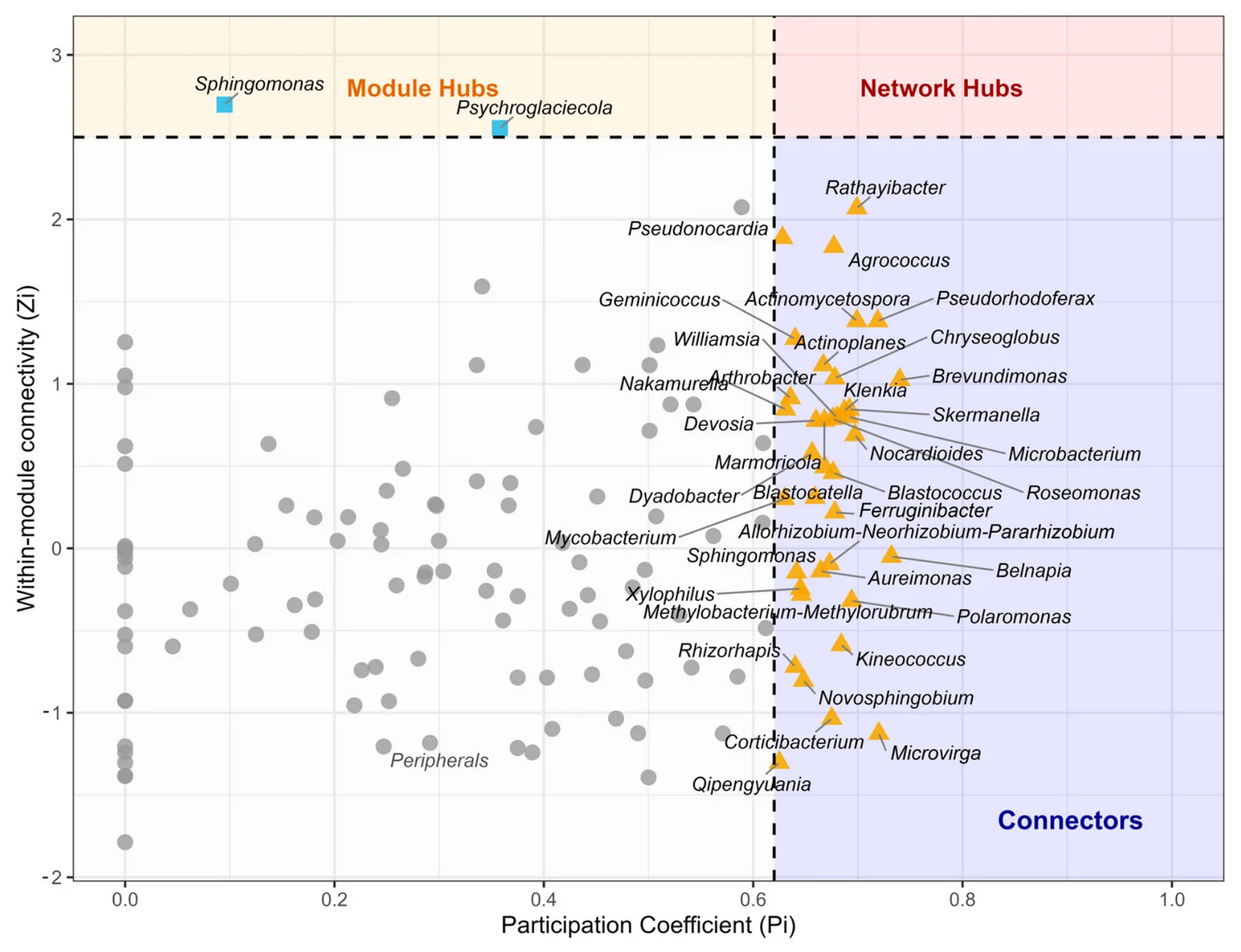
Network topology and topological roles of the bacterial community in male *H. lusitanicum* ticks. Methodological framework, coordinate axes (Z_i_ and P_i)_, and role threshold boundaries are identical to those described in Figure 9.

To evaluate the functional and structural importance of specific bacterial taxa within the *H. lusitanicum* microbiome, we calculated the within-module connectivity (Z_i_) and participation coefficient (P_i_) for all nodes across both sex-specific networks. Distinct structural organizations and degrees of centralization emerged between the two sexes (Figure 9 and Figure 10).

**Figure 11:**
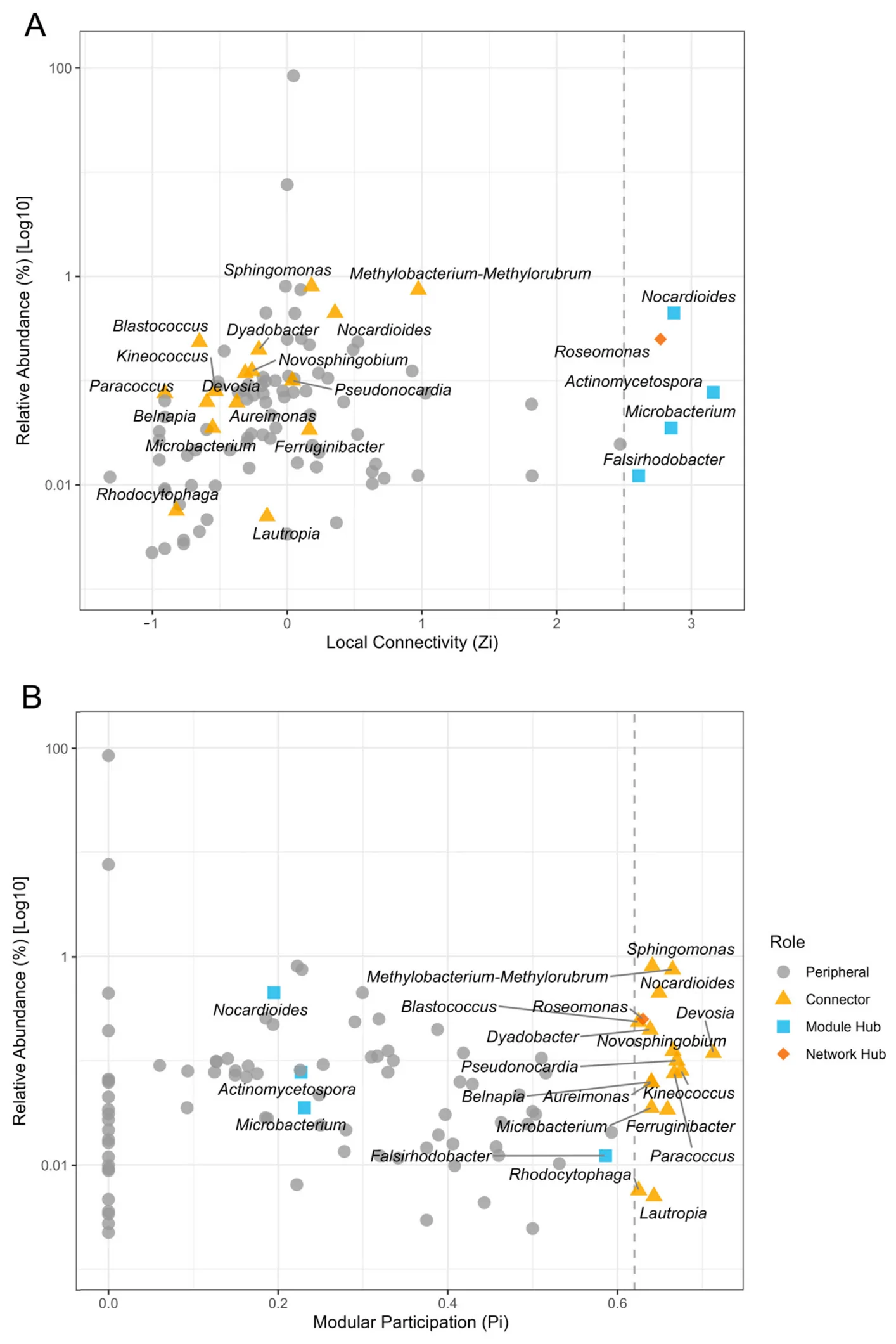
Microbiome topological roles versus mean relative abundance profiles in female *H. lusitanicum* ticks. The dual-panel plot integrates network-derived node parameters with log-transformed mean relative abundances (%). (**A**) Distribution of bacterial genera across the local within-module connectivity axis (Z_i_). (**B**) Distribution of bacterial genera across the modular participation coefficient axis (P_i_). In both panels, the vertical dashed lines indicate the mathematical thresholds (Z_i_ = 2.5 and P_i_ = 0.62, respectively) used to categorize network roles. Point shapes designate specific topological classifications (circle: peripheral; triangle: connector; square: module hub; rhombus: network hub). Highly central or abundant taxa are explicitly labeled to highlight the distribution of key microbial anchors against their cumulative baseline abundance. Vertical dashed line represents Zi threshold (**A**) or P_i_ threshold (**B**).

Mapping genus abundance against topological roles (Figure 11) confirmed a striking decoupling between numerical dominance and network centrality. While the most abundant taxa clustered exclusively as peripheral nodes, key structural roles were maintained by low- to intermediate-abundance genera. In the local connectivity space (Figure 11A), *Roseomonas* emerged as a distinct Network Hub, while *Nocardioides*, *Actinomycetospora*, *Microbacterium*, and *Falsirhodobacter* acted as Module Hubs regulating local sub-communities. Concurrently, the modular participation space (Figure 11B) identified an active guild of intermediate-abundance connectors, led by *Sphingomonas*, *Nocardioides*, *Devosia*, and *Novosphingobium*, which bridge discrete ecological modules across the female microbial co-occurrence network.

**Figure 12:**
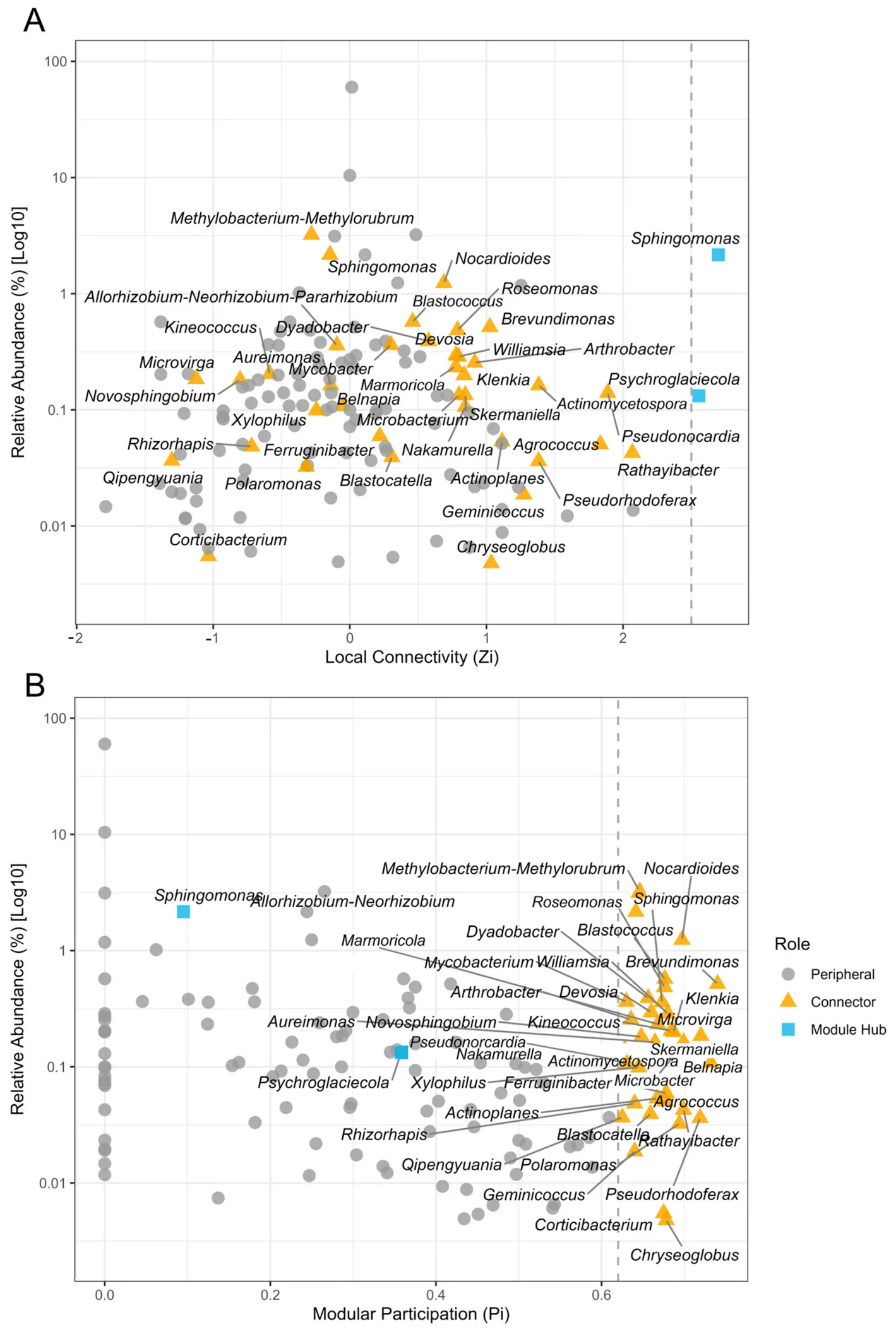
Microbiome topological roles versus mean relative abundance profiles in male *H. lusitanicum* ticks. This dual-panel plot follows the exact mathematical thresholds (Z_i_ = 2.5; P_i_ = 0.62), log-transformations, and geometric coding described in Figure 11. Labeled taxa highlight the absence of global leaders (network hubs) and the extensive expansion of inter-module connectors (triangles) and local coordinators (module hubs; squares) that sustain the dense architectural cohesion of the male tick microbiome. Vertical dashed line represents Zi threshold (**A**) or P_i_ threshold (**B**).

In male ticks (Figure 12), the interplay between abundance and topology shifted toward a decentralized framework. Mirroring the females, numerically dominant taxa remained peripheral. However, the male taxonomic association network completely lacked global Network Hubs. Instead, local architecture was maintained by only two Module Hubs: *Sphingomonas,* exhibiting a remarkably high relative abundance, and the low-abundance genus *Psychroglaciecola* (Figure 12A). Strikingly, the modular participation space (Figure 12B) revealed a massive, high-density constellation of intermediate- and low-abundance connectors (including *Nocardioides*, *Methylobacterium-Methylorubrum*, *Blastococcus*, *Brevundimonas*, and *Devosia*, among others) crossing the participation threshold, underscoring an extensively integrated inter-module traffic.

### 3.5. Topological Robustness and Vulnerability to Targeted Attacks

**Figure 13:**
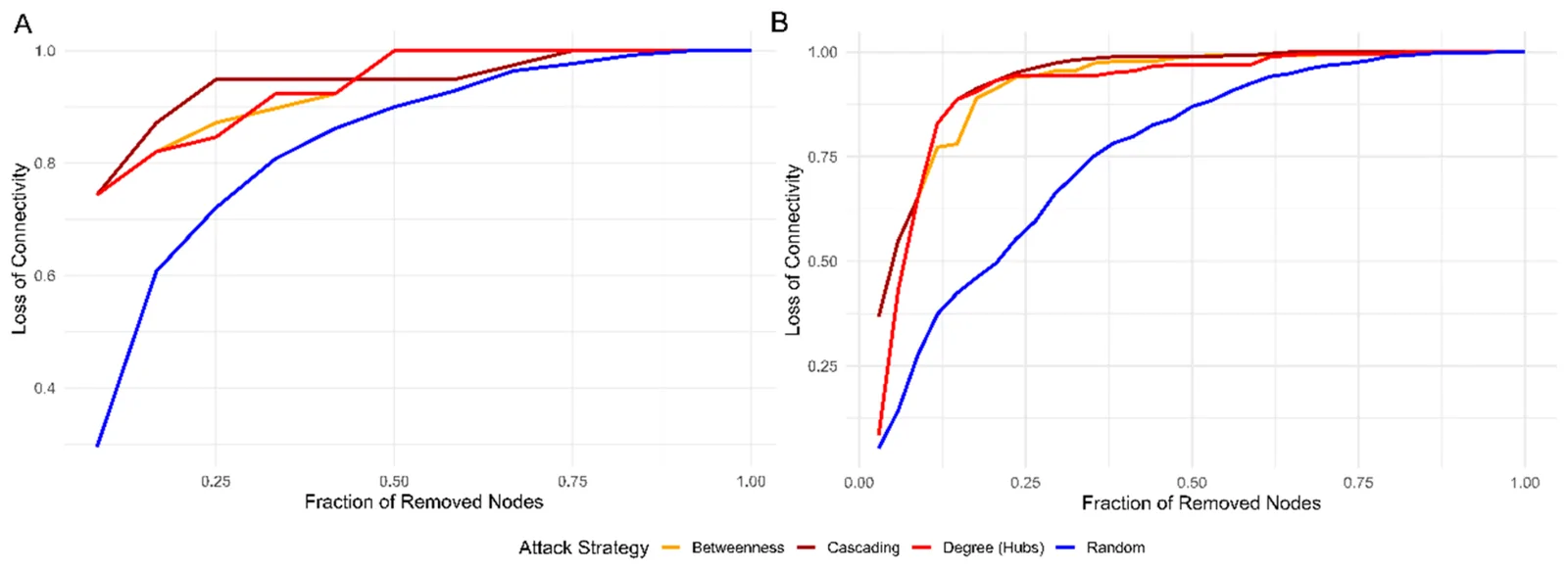
Topological robustness and vulnerability analysis of the *H. lusitanicum* core microbiome networks. Curves illustrate the loss of network connectivity (*Y*-axis) relative to the fraction of nodes removed (*X*-axis) under four distinct degradation scenarios: random failure (blue) and targeted attacks driven by node betweenness (yellow), cascading effects (dark red), and node degree (red). (**A**) Female network vulnerability profile, displaying an immediate structural collapse upon targeted keystone removal. (**B**) Male network vulnerability profile, demonstrating higher resilience to random microbial loss but acute sensitivity to the targeted removal of its functional network Connectors.

To evaluate the structural stability and resilience of the *H. lusitanicum* core taxonomic association networks, we simulated network degradation under both random failures and targeted attacks (removal of nodes based on degree, betweenness, and cascading centrality) (Figure 13). Both sex-specific networks exhibited a classic scale-free behavior, showing higher tolerance to random node loss than to targeted keystones removal. However, a stark divergence in vulnerability profiles was observed. The female network demonstrated an immediate, brittle collapse under targeted attacks; the removal of the initial fraction of high-centrality nodes caused an instantaneous 75% loss of overall connectivity, reflecting an ecosystem critically dependent on a few discrete taxonomic anchors. Conversely, the male network exhibited significantly greater buffering capacity against random perturbations (blue curve), maintaining higher topological cohesion under gradual random loss. Nevertheless, the male network was highly sensitive to targeted attacks on its structural brokers, experiencing a sharp, precipitous drop in connectivity when betweenness- and degree-prominent Connectors were systematically eliminated.

## 4. Discussion

Comprehensive macro-microbial community analyses are critical to deciphering tick biology, given that they harbor complex assemblages of potentially pathogenic and non-pathogenic endosymbionts [64]. These microbial partners act as key determinants of tick evolutionary success, directly driving processes such as metabolic adaptation, survival, competence for pathogen transmission, immune homeostasis, and reproductive fitness [65,66,67]. The tick microbiome is shaped by a complex interplay of environmental filters, host biological traits, and maternally derived vertical transmission. While maternal legacy represents a foundational driver of the internal microbiota, its relative contribution may also depend on the tick’s physiological state, distinguishing fully engorged reproductive females from the unfed, questing adults evaluated here.

While high-throughput sequencing surveys frequently identify non-endosymbiotic bacteria as low-abundance, transient constituents of the tick microbiome, the internal ecosystem is invariably dominated by obligate endosymbionts. Genera such as *Coxiella*, *Francisella*, *Rickettsia*, and *Midichloria* represent the omnipresent pillars of these microbial communities [68,69]. Characterized by an intracellular lifestyle and strict maternal inheritance, these symbionts possess markedly reduced genomes relative to pathogenic species within the same genera. Although this genomic erosion suggests an evolutionary transition from a pathogenic ancestor, whether these core endosymbionts retain any cryptic pathogenic potential or virulence factors remains to be elucidated.

Our multifactorial PERMANOVA model demonstrates that the microbial assembly of *H. lusitanicum* is framed by both biogeographical filters and host biological traits, which independently account for a substantial portion of the total community variation (*R*^2^ = 0.111 and *R*^2^ = 0.095, respectively). The global model accounted for 24.3% of the total bacterial community variation (*R*^2^ = 0.243, *p* = 0.001). When partitioning this variance, the significant effect of the ecological sampling site (*R*^2^ = 0.111, *p* = 0.001) underscores the role of environmental acquisition and local microclimatic conditions in modulating the tick’s transient microbiome. Variations in local vegetation, relative humidity, and the specific composition of the local vertebrate host community across different localities likely dictate the pool of environmental bacteria to which *H. lusitanicum* is exposed, driving localized shifts in alpha diversity, such as the significant richness contrasts observed between Cazorla, Andujar, and Antequera.

A limitation of the present study is the unbalanced sampling design across sexes and geographic locations, driven by the natural scarcity of questing individuals in certain habitats (e.g., Antequera). Because male ticks exhibit distinct microbial diversity profiles compared to females, sex imbalance can potentially confound spatial comparisons. To mitigate this bias, locality-level diversity analyses were stratified by sex, confirming that geographic variation in the microbiome persists independently of sex ratio skewing. Nevertheless, future studies with expanded and balanced sampling schemes will be valuable to further disentangle local environmental drivers from sex-specific microbiome dynamics.

Crucially, the non-significant interaction between host sex and locality (*R*^2^ = 0.037, *p* = 0.470) reveals that the compositional divergence between male and female microbiomes is a geographically robust phenomenon, preserved across distinct ecogeographical constraints. When evaluating this macro-topological partition through the lens of network theory, this spatial consistency aligns perfectly with our discovery of a hardwired, topologically invariant core shared across both sexes at the strict sequence level. According to the reference inventory, seven distinct ASVs consistently maintained their exact roles as vital inter-module connectors in both male and female networks: *Aureimonas* (ASV_050), *Belnapia* (ASV_161), *Devosia* (ASV_442), *Dyadobacter* (ASV_021), *Ferruginibacter* (ASV_225), *Novosphingobium* (ASV_381), and *Sphingomonas* (ASV_087). The persistence of these identical sequences across sex-specific boundaries demonstrates that their role as ecological “bridges” is a foundational, intrinsic property of the *H. lusitanicum* core microbiota, likely maintaining essential homeostatic pathways regardless of geographical origin or host sex.

A deeper taxonomic inspection revealed an asymmetrical strategy in microbiome stability and individual uniformity between sexes, beginning with a stark architectural contrast in females. Female ticks exhibited a highly conserved, streamlined, and uniform bacteriome profile, rigidly dominated by the primary obligate endosymbiont *Francisella* (constituting 60% to 90% of total abundance) and the secondary co-symbiont *Candidatus* Midichloria, with minimal background variation from other bacterial groups [62,70,71]. Structurally, this closed, high-dominance ecosystem provides the ecological rationale for the centralized, “top-down” network architecture observed in the female community (384 nodes, 1578 edges).

Application of the Zi-Pi topological framework revealed that this network is anchored by a highly centralized global keystone, a feature unique to the female microbiome, identified as *Roseomonas* (ASV_051). In ecological network theory, network hubs act as critical global moderators that bind separate modules into a cohesive whole, coordinating systemic functions across the entire ecosystem. While this configuration ensures highly coordinated communication across modules under stable conditions through a tight, non-redundant pipeline of 24 connectors, it exposes a major structural vulnerability. The reliance of the female microbial community on a solitary global moderator suggests that the system may be highly susceptible to catastrophic fragmentation or dysbiosis if this specific *Roseomonas* hub is disrupted.

Conversely, host sex independently exerted a comparable selective pressure (*R*^2^ = 0.095, *p* = 0.001), shaping a fundamentally different internal niche in male individuals. This sex-specific divergence is highly consistent with the known biology of hard ticks, where distinct questing behaviors, varying blood-meal digestion dynamics, and dimorphic immune or endocrine responses allow for a more permissive colonization. Consequently, male ticks presented a significantly higher alpha diversity (*p* < 0.001) and an increased degree of inter-individual heterogeneity, characterized by a plastic pattern where diverse environmental and transient lineages, such as *Acinetobacter*, *Sphingomonas*, *Stenotrophomonas*, and *Brevundimonas*, accumulate in the basal fraction of the microbiome. Remarkably, these quantitative asymmetries extend down to strict sequence-level variations within the primary endosymbiont itself; while *Francisella*_ASV1 established the foundational baseline across all individuals, *Francisella*_ASV3 exhibited a strict, sex-biased distribution restricted exclusively to the top 50 framework of male ticks, highlighting a layer of genetic micro-heterogeneity. The notable diversity of *Francisella* strains identified in this study (Table A3) suggests a complex evolutionary history within *H. lusitanicum*. Rather than stemming from a single ancestral colonization event, this microdiversity is best explained as a consequence of multiple independent acquisition events, likely driven by horizontal transmission or environmental exposure throughout the tick’s evolutionary trajectory [62,72]. In this context Krawczyk et al. (2022) [73], proposed that *Midichloria mitochondrii* alters the propensity of ticks to acquire or maintain horizontally acquired pathogens. The authors argued that these remarkable interactions are governed by a complex web of positive and negative associations operating both between and within horizontally and vertically transmitted symbionts, highlighting underlying physiological mechanisms that merit further investigation.

This permissive and fluctuating bacterial pool provides the ecological rationale for a massive male network expansion (492 nodes, 6501 edges) and a complete reorganization of its structural principles. Entirely lacking global Network Hubs, the male microbiome compensates for the absence of a global leader through an incredibly dense architecture, characterized by a higher clustering coefficient (0.500 vs. 0.361 in females) and a decentralized “distributed control” mechanism. This structural robustness is driven by a massive expansion of Connectors, recruiting nearly three times as many taxa (68 ASVs) as the female network (24 ASVs). This multi-communitarian pipeline in males, dominated by multiple highly versatile and redundant ASVs from genera like *Nocardioides*, *Sphingomonas*, and *Actinomycetospora*, functions as a vital buffer for metabolic and information flow. This web of redundant connectors provides functional insurance: if individual taxa fluctuate due to host physiological shifts, alternative pathways remain available, effectively preventing localized dysbiotic expansions, such as the sporadic *Acinetobacter* or *Brevundimonas* overgrowths captured in individual wild specimens, from destabilizing the essential homeostatic core of the holobiont.

Beyond the static classification of taxa, our comparative analysis unveiled a fascinating phenomenon of cross-sex topological plasticity involving key mutual actors. Rather than maintaining rigid ecological roles, specific core bacteria dynamically repurposed their network influence in response to the sex-specific landscape. Most notably, *Roseomonas* (ASV_051) dynamically transitioned from its role as a critical, high-risk global Network Hub in the strict female network to a flexible inter-module Connector within the dense, heterogeneous male ecosystem. Concurrently, *Nocardioides* (ASV_065) and others companion bacterial lineages, shifted from a local Module Hub in females to a decentralized Connector in males.

The topological prominence of environmental and secondary genera, such as *Roseomonas* and *Nocardioides*, within our co-occurrence networks aligns with recent microbial profiling in ixodid ticks. *Roseomonas* strains, including newly described tick-associated species like *Roseomonas haemaphysalidis* [36,37,74], have been successfully isolated from both wild tick populations and their larval progeny (e.g., in *Dermacentor* spp.), validating their persistence across tick life cycles. Similarly, *Nocardioides* is frequently recovered in high-throughput 16S rRNA surveys as a prominent constituent of the non-endosymbiotic microbiome acquired from soil or host skin habitats [73]. Our network backbones elevate these findings by demonstrating that these environmentally derived taxa are not transient contaminants; rather, they are functionally integrated into the core microbial community architecture, acting as key connectors or topological hubs that stabilize the tick’s internal microbial ecosystem.

Integrating the topological-abundance landscapes of both sexes highlights two fundamentally divergent evolutionary strategies for microbiome organization in *H. lusitanicum*. In females (Figure 9 and Figure 11), the system relies on a strict ‘top-down’ hierarchy where a single, low-abundance Network Hub (*Roseomonas*) and discrete Module Hubs orchestrate community communication, creating a centralized but structurally brittle network. Conversely, the male microbiome (Figure 10 and Figure 12) bypasses global hubs entirely, transitioning into a highly cooperative, distributed network. The massive constellation of connectors in males suggests that metabolic and physiological demands are met through a coordinated, multi-taxa pipeline. In this male-specific framework, intermediate-abundance genera like *Sphingomonas* act as local facilitators rather than monopolies, working alongside an extensive guild of low-abundance environmental and secondary brokers. This sexual dimorphism demonstrates that while female ticks maintain ecosystem control through centralized keystones, male ticks rely on topological redundancy and widespread inter-module collaboration to preserve microbiome homeostasis.

This evolutionary flexibility suggests that these core microbial anchors are not structurally static; instead, they possess the functional versatility to shift from local coordinators to global mediators. This adaptive rewiring allowed them to maintain systemic cross-talk and buffer the holobiont, successfully navigating the distinct physiological demands, hormonal environments, and reproductive constraints that characterize male and female *H. lusitanicum* ticks in the wild.

Our topological findings build upon and significantly expand previous baseline characterizations of the *H. lusitanicum* bacteriome [70,75]. Prior studies utilizing high-throughput sequencing have predominantly focused on profiling the taxonomic composition and diversity of this tick’s microbiome, consistently identifying a community structured around maternal endosimbionts alongside fluctuating secondary symbionts and environmental taxa [76].

A growing body of literature has established that primary and secondary endosymbionts, including *Rickettsia*, *Candidatus Midichloria*, *Coxiella*, and *Francisella*, constitute the core functional bacteriome of hard ticks, playing non-redundant, pivotal roles in host fitness and physiological homeostasis [77,78,79]. Our network topology contextualizes these long-standing symbioses within a community-wide framework. While several authors have characterized these maternal endosymbionts as isolated metabolic pillars, our interactome data suggests they may act as high-centrality anchors or driving forces behind community structure. Specifically, the strict, top-down centralized control observed in females might be orchestrated by the overwhelming physiological dominance of these specialized endosymbionts, which limits peripheral cross-talk via niche exclusion. Conversely, the transition to a decentralized, hyper-connected pipeline in males may reflect a metabolic shift where secondary or environmental taxa form cooperative consortia to supplement or interact with these primary symbionts, fulfilling different nutritional demands dictated by host sex.

However, by transitioning from mere taxonomic inventories to a topological co-occurrence network framework, our analysis uncovers a layer of biological complexity previously overlooked in *H. lusitanicum*: a stark sexual dimorphism in microbial cross-talk. While previous literature documented differences in bacterial abundance between sexes, our data contextualizes these shifts ecologically. The centralized, top-down structure in females suggests that specific keystone taxa, such as the *Roseomonas* (ASV_051) Network Hub, exert a strict niche exclusion, whereas the hyper-dense, cooperative backbone in males reflects a highly coordinated metabolic pipeline. Consequently, these results demonstrate that host sex does not merely alter microbial census, but fundamentally rewires the ecological rules and cooperative dynamics governing the *H. lusitanicum* microbiome.

To evaluate the static structural cohesion of the co-occurrence graphs, we performed targeted and random node removal simulations. Rather than modeling dynamic biological extinction cascades—which in living systems are buffered by ecological rewiring—this analysis measures the passive topological tolerance of the network architecture. These simulations highlight distinct structural trade-offs shaping the *H. lusitanicum* bacteriome across host sexes. The higher sensitivity of the female network to targeted node removal reflects the topological characteristics of a highly centralized, ‘top-down’ architecture. In females, graph cohesion relies heavily on dominant structural anchors (such as *Roseomonas*); removing these primary hubs rapidly disconnects peripheral nodes from the main network component. In contrast, the decentralized network topology characteristic of the male microbiome provides greater structural redundancy. The more gradual loss of connectivity observed in males under progressive node removal reflects a distributed control architecture sustained by a broad array of inter-module connectors. While natural microbial communities can dynamically reorganize and rewire following environmental perturbations, these baseline static topologies demonstrate how host sex fundamentally dictates the structural organization and baseline topological cohesion of the vector microbiome.

Furthermore, when interpreting the taxonomic variability observed in non-engorged ticks, it is essential to consider the concept of functional redundancy. From a holobiont perspective, tick physiological homeostasis depends primarily on the metabolic output provided by the microbial community rather than strict taxonomic fidelity. Consequently, the high bacterial diversity and variable network topologies identified in questing adults, particularly males, may reflect interchangeable taxa occupying equivalent functional niches (e.g., nutrient biosynthesis, stress adaptation). Future research utilizing shotgun metagenomics or metatranscriptomics will be key to directly mapping these functional pathways without relying on 16S-based predictive algorithms, which remain constrained by reference database limitations in non-model vector species.

## 5. Conclusions

The microbiota of *Hyalomma lusitanicum* is shaped by a dual framework where ecogeographical location (*R*^2^ = 0.111) and host biological sex (*R*^2^ = 0.095) independently govern community assembly. Notably, this sex-linked divergence remains geographically consistent across capture sites, reflecting not only compositional shifts but also fundamental differences in microbiome stability, characterized by a highly conserved core in females versus greater compositional variability in males.

Host sex governs the individual stability and permissive threshold of the tick microbiome. Females maintain a highly streamlined, uniform, and strict endosymbiotic profile overwhelmingly dominated by *Francisella* and *Candidatus Midichloria*, whereas males present a highly heterogeneous and permissive landscape that allows a diverse influx of environmental companion lineages (*Acinetobacter*, *Sphingomonas*, *Stenotrophomonas*, *Brevundimonas*) to accumulate in the basal fraction.

These distinct taxonomic profiles translate into divergent macro-topological network strategies: the strict, closed female microbiome relies on a centralized, ‘top-down’ structure organized around a single global Network Hub (*Roseomonas* ASV_051), while the variable, open male microbiome achieves systemic robustness through a highly dense (6501 edges), decentralized network reinforced by a massive redundant pipeline of 68 inter-module connectors.

Despite these sex-specific variations, a hardwired and topologically invariant core of seven identical ASVs (belonging to *Aureimonas*, *Belnapia*, *Devosia*, *Dyadobacter*, *Ferruginibacter*, *Novosphingobium*, and *Sphingomonas*) operates as a universal functional backbone across both networks, maintaining systemic cross-talk and holobiont integrity regardless of the host’s sex or geographical origin.

## Figures and Tables

**Table 1:** Demographic composition of collected ticks. The table shows the total count between males and females.

Locality	Females	Males	Total
Andujar	7	13	20
Mancha Real	9	11	20
Cazorla	7	1	8
Tabernas	9	3	12
Lantejuela	10	9	19
Antequera	7	0	7
**Total**	49	37	86

## Data Availability

The raw sequencing data are currently pending assignment of a BioProject accession number, which will be provided prior to publication. All custom code, scripts, and processed data matrices required to reproduce the analyses and figures presented in this manuscript are freely available on GitHub (v. 2 55.0) at: https://github.com/jmarquez20/Tick_microbiome_Hlusitanicum_2026 (accessed on 30 July 2026). Data are available from the authors upon reasonable request.

## Appendix A

**Table A1:** Conceptual framework translating network topological metrics into biological and ecological roles within the tick microbiome. Definition, mathematical criteria (Z_i_ and P_i_ parameters), and ecological interpretations of topological roles identified in the co-occurrence networks of *Hyalomma lusitanicum*.

Topological Role	Mathematical Criteria	Ecological Definition and Biological Interpretation
Module	Densely connected sub-graph	Ecological Sub-community: A cluster of bacterial taxa that closely co-occur or share a specific micro-environment/functional niche within the tick host.
Peripheral nodes	Z_i_ ≤ 2.5 and P_i_ ≤ 0.62	Module specialists: Taxa with few links inside or outside their module. They represent transient, environmental, or highly specialized bacteria with low global structural influence.
Module hubs	Z_i_ > 2.5 and P_i_ ≤ 0.62	Local coordinators: Taxa with high connectivity inside their own module but few connections to others. They drive local ecological cohesion and metabolic interactions within their specific sub-community.
Connectors	Z_i_ ≤ 2.5 and P_i_ > 0.62	Biological bridges: Taxa that link different, otherwise isolated modules. They act as cross-talk mediators, facilitating the exchange of metabolites or signals across sub-communities and maintaining overall network cohesion.
Network hubs	Z_i_ > 2.5 and P_i_ > 0.62	Keystone Taxa/Master Organizers: Global structural anchors highly connected both within and across modules. Their presence underpins system stability; their disruption can trigger community-wide restructuring.

**Table A2:** Permutation test results for homogeneity of multivariate dispersions (*betadisper*) across host sex and sampling locality based on Bray–Curtis’s dissimilarity.

Factor	Source of Variation	Df	Sum of Squares	Mean Squares	*F*-Statistic	Permutations	*p*-Value (Pr (>*F*))
Sex	Groups	1	75.906	75.906	31.551	999	<0.001 ***
	Residuals	84	202.093	2.406	—	—	—
Locality	Groups	21	86.037	40.970	1.3204	999	0.248 (ns)
	Residuals	64	198.588	31.029	—	—	—

Note: Df, degrees of freedom; ns, not significant (*p* ≥ 0.05). Significance codes: *** *p* < 0.001. Permutation tests were executed using 999 free iterations with the vegan R package. A significant *p*-value for tick sex indicates unequal multivariate dispersion (heteroscedasticity) between male and female ticks, whereas non-significant variation across sampling localities confirms the assumption of multivariate dispersion homogeneity.

**Table A3:** Sequence metrics, taxonomic assignments, and NCBI GenBank repository accessions for the core Amplicon Sequence Variants (ASVs) resolved in *H lusitanicum* ticks. Columns detail the designated ASV identifier, total read counts, nucleotide sequence length (bp), GenBank accession numbers with their corresponding percentage of sequence identity, and the closest taxonomic description.

ASV	Counts	Length	Accession (% of Identity)	Description
ASV1	2,761,147	427	MW131669.1 (100%); MZ605211.1 (100%)	*Francisella* endosymbiont of *Hyalomma lusitanicum* isolate Loc3b & *Francisella* endosymbiont of Dermacentor auratus isolate FL14
ASV3	23,770	427	MW131669.1 (99.766%); MZ605211.1 (99.766%)	*Francisella* endosymbiont of *Hyalomma lusitanicum* isolate Loc3b & *Francisella* endosymbiont of *Dermacentor auratus* isolate FL14
ASV7	8535	427	EU332821.1 (100%); MN998636.1 (100%)	*Francisella*-like endosymbiont of *Amblyomma* sp. 16S & Uncultured *Francisella* sp. clone FraAhume1
ASV11	3446	427	MZ605210.1 (99.766%); OR508970.1 (99.766%)	*Francisella* endosymbiont of *Dermacentor auratus* isolate FL16 & *Francisella*-like endosymbiont isolate 1ftsi
ASV36	3388	427	MZ605210.1 (99.766%); OR508970.1 (99.766%)	*Francisella* endosymbiont of *Dermacentor auratus* isolate FL16 & *Francisella*-like endosymbiont isolate 1ftsi
ASV37	3334	427	MN998650.1 (100%); MW287940.1 (100%)	Uncultured *Francisella* sp. clone FraAscul & *Francisella*-like endosymbiont of *Amblyomma sculptum* isolate AmbscuOcaj1
ASV38	3013	427	MZ605210.1 (99.766%); OR508970.1 (99.766%)	*Francisella* endosymbiont of *Dermacentor auratus* isolate FL16 & *Francisella*-like endosymbiont isolate 1ftsi
ASV43	2994	427	MZ605210.1 (99.766%); OR508970.1 (99.766%)	*Francisella* endosymbiont of *Dermacentor auratus* isolate FL16 & *Francisella*-like endosymbiont isolate 1ftsi
ASV44	2894	427	MZ605210.1 (99.766%); OR508970.1 (99.766%)	*Francisella* endosymbiont of *Dermacentor auratus* isolate FL16 & *Francisella*-like endosymbiont isolate 1ftsi
